# Phenotypic Similarities in Flower Characteristics Between Novel Winter-Hardy Hibiscus Hybrids and Their Tropical Relatives

**DOI:** 10.3389/fpls.2019.01528

**Published:** 2019-11-22

**Authors:** Dariusz P. Malinowski, William E. Pinchak, Kristin Yanker-Hansen

**Affiliations:** ^1^Texas A&M AgriLife Research and Extension Center, Texas A&M University System, Vernon, TX, United States; ^2^California Horticultural Society, San Francisco, CA, United States

**Keywords:** *Hibiscus moscheutos*, *Hibiscus rosa-sinensis*, *Hibiscus syriacus*, Malvaceae, ornamental plants, temperate environments

## Abstract

Herbaceous winter-hardy *Hibiscus* spp. in the section *Muenchhusia*, also known as rosemallows, are attractive ornamental plants found in temperate environments. These should not be confused with woody winter-hardy hibiscus (*Hibiscus syriacus* L. and related species) which have also been intensively used as ornamental shrubs. During the past 70 years, breeders have attempted to create winter-hardy hibiscus hybrids with novel flower colors resembling the distantly related tropical Chinese hibiscus, *Hibiscus rosa-sinensis* L. Although direct attempts to hybridize winter-hardy hibiscus with the tropical hibiscus have been unsuccessful, new interspecific herbaceous winter-hardy hibiscus hybrids with a palette of novel flower colors commonly found in tropical hibiscus have been recently introduced. In this review, we outline the historic perspective on interspecific hybridizations in woody and herbaceous winter-hardy hibiscus and discuss breeding approaches to develop herbaceous winter-hardy hibiscus hybrids with novel flower colors and shapes resembling tropical hibiscus cultivars. By creating a broad genetic variability in herbaceous winter-hardy hibiscus hybrids we found a successful approach to increase the range of flower colors and shapes in these species and made them look very like their distant tropical relatives.

## Introduction

The genus *Hibiscus* contains over 300 annual and perennial species belonging to the family Malvaceae (Pfeil et al., 2002; Akpan, 2007) that are among the oldest flowering plants on Earth (Wang, 2010). *Hibiscus* species grow on all continents except for Antarctica and have adapted to environments ranging from temperate to tropical. It has been speculated that all *Hibiscus* species evolved from a common ancestor that appeared about 135 million years ago, at the time of Gondwana that broke up from Pangea about 180 million years ago (Fryxell, 1965; Hawkes and Smith, 1965; Fryxell, 1967; Black, 2017). Individual *Hibiscus* species grow as herbaceous plants, shrubs or trees (Winters, 1970; Fryxell, 1980; Akpan, 2007; Hinsley, 2009). Almost all *Hibiscus* species have showy flowers. Although the appearance of their flowers may vary, the structure of the flower is similar in all *Hibiscus* species and consists of five petals, five-toothed staminal column apex, styles usually branching at the apex, stigmas usually terminal, style branches equal in number to the carpels, a fruit capsule, and absent gossypol glands (Pfeil et al., 2002). Except for the tropical Chinese hibiscus, which has been extensively cultivated and hybridized for centuries, the most common flower colors within other *Hibiscus* species are usually yellow, white, pink, or red (Lawton, 2004). In contrast, the tropical Chinese hibiscus has now thousands of cultivars with a huge range of colors and color combinations, including blue (International Hibiscus Society, 2019).

The few species that are very popular as perennial ornamental garden plants with showy flowers and adapted to temperate environments are scarlet rosemallow (*Hibiscus coccineus* Walter), crimson-eyed rosemallow (*Hibiscus moscheutos* L.), rose of sharon (*Hibiscus syriacus* L.), and confederate rose (*Hibiscus mutabilis* L.) (Lawton, 2004). Their flowers range from red (rarely white) in scarlet rosemallow, to white, pink, and red in crimson-eyed rosemallow, white, pink, lavender, and blue in rose of sharon, and white changing to pink in confederate rose. In recent years, the Texas A&M AgriLife Research hibiscus breeding program has developed many *H.* x *moscheutos* hybrids with novel flower colors (Malinowski et al., 2012a; Malinowski et al., 2012b; Ledbetter, 2017). The first cultivars (patents pending) from this program with bluish, purple, coral, magenta, and other colors heretofore absent in *H. moscheutos* were introduced in the USA in by J Berry Nursery in collaboration with Texas A&M AgriLife Research as the Summer Spice Hibiscus™ series in 2018 (Fannin, 2018).

In this review, we outline the historic perspective on interspecific hybridizations among *Hibiscus* species to create hybrids with unique, showy flowers adapted to temperate climatic zones. Specifically, we present examples of novel flower trait expressions in complex *H.* x *moscheutos* hybrids that resemble phenotypic flower traits observed in modern hybrids of the tropical Chinese hibiscus. We propose that further intensive hybridization of *Hibiscus* species within the section *Muenchhusia* may be a valuable avenue to create herbaceous winter-hardy hibiscus hybrids with tropical-looking flowers and novel flower colors and shapes similar to those achieved in the tropical Chinese hibiscus in the past few hundred years of breeding. The winter-hardy hibiscus hybrids are described here only to show similarities in their phenotypic flower traits to the tropical Chinese hibiscus.

## Interspecific Hybridization Among *Hibiscus* Species—Successes and Pitfalls

Inter- and intra-specific hybridization among different species and genera is one of the most important breeding methods for creating new and improved ornamental plants (Van Tuyl and De Jeu 1997; Kuligowska et al., 2016). Breeders, both professional and amateurs, have been trying to create interspecific hybrids between winter-hardy (herbaceous and woody) hibiscus species and the tropical Chinese hibiscus to create “tropical”-looking hibiscus hybrids adapted to temperate environments; however, the attempts have been unsuccessful (Yu et al., 1976; Paek et al., 1989; Kuligowska et al., 2013). The historic perspective on interspecific hybridization in winter-hardy hibiscus species is presented in **Table 1**. The major problem with hybridizing winter-hardy with tropical *Hibiscus* species is their genetic incompatibility. Herbaceous winter-hardy species belonging to the section *Muenchhusia* and native to North America (*H. coccineus*, *H. moscheutos*, *Hibiscus militaris*, *Hibiscus dasycalyx*, and *Hibiscus lasiocarpos*) have 2*x* = 2*n* = 38 chromosomes (Hinsley, 2012). The chromosome numbers in the tropical *H. rosa-sinensis* group vary and may be (2*n* = ) 36, 38, 40, 44, 46, 52, 70, 76, 84, 90, 92, 118, 132, or 144, depending on the ecotype and degree of hybridization. In addition, numerous pre- and postfertilization barriers prevent interspecific hybridization and/or production of fertile F_1_ plants in *Hibiscus* species belonging to different sections (Hogenboom, 1973; Van Tuyl and De Jeu, 1997; Van Laere et al., 2007). In contrast, *H. rosa-sinensis* hybridizes easily with other *Hibiscus* species belonging to the section Lilibiscus (Thomson and Braglia, 2019).

**Table 1:** Historic perspective of major accomplishments in development of winter-hardy interspecific hibiscus hybrids.

Year	Achievement	Reference
1964	“Ai-Fuyo” amphidiploid hybrid between *H. mutabilis* and *H. moscheutos*	Kuwada (1964)
1976	Sterile seeds were obtained from an interspecific hybridization between *H. syriacus* and *H. rosa-sinensis*	Yu et al. (1976)
1989	Attempts to create somatic hybrids between *H. syriacus* and *H. rosa-chinensis* through protoplast fusion	Paek et al. (1989)
2001	Development of interspecific hybrids between *H. syriacus or H. sinosyriacus* and *H. paramutabilis*	Kyung and Kim (2001)
2003-2012	Release of “Angelique”, “Pink Comet”, “Cherub”, and “Satellite” alleged interspecific hybrids between *H. moscheutos* (or *H. moscheutos* x *H. coccineus*) and *H. rosa-sinensis*	United States Plant Patent (2003a, 2003c; 2006; 2012)
2007	Development of interspecific hybrids between *H. syriacus* and *H. paramutabilis*, and *H. syriacus and H. sinosyriacus*	Van Laere et al. (2007)
2013	Analysis of incompatibility reactions in interspecific crosses between *H. rosa-sinensis* and *H. syriacus* or *H. moscheutos*	Kuligowska et al. (2013)
2016	Detection of pre- and post-fertilization hybridization barriers in crosses among *Hibiscus* species in the *Muenchhusia* section, e.g., *H. coccineus*, *H. laevis* and *H. moscheutos*.	Kuligowska et al. (2016)
2016	“Hapa White”, “Hapa Red”, and “Hapa Pink” interspecific hybrids between *H. mutabilis* and *H. moscheutos*	Pounders and Sakhanokho (2016)

Breeders have successfully created polyploid hybrids between the temperate winter-hardy *Hibiscus* species rose of sharon (and its closely related species *Hibiscus sinosyriacus* Bailey) and *Hibiscus paramutabilis* Bailey with the goal to increase flower size and extend the blooming period of *Hibiscus syriacus* and *H. sinosyriacus* (Eeckhaut, 2003). These woody *Hibiscus* species are native to China (Bates, 1965). Both *H. syriacus* and *H. sinosyriacus* are similar morphologically and genetically, but different from *H. paramutabilis* (Van Huylenbroeck et al., 2000). Chromosome numbers are 2*x* = 2*n* = 80 for *H. syriacus* and *H. sinosyriacus*, and 2*x* = 2*n* = 82 for *H. paramutabilis* (Niimoto, 1966). The breeding efforts have resulted in several unnamed hybrid experimental lines (Van Huylenbroeck et al., 2000; Kyung and Kim, 2001; Kyung et al., 2001; Eeckhaut et al., 2004) and cultivars, i.e., Daewangchun (Ha et al., 2015); Tahagol Red (Ha et al., 2014), and Woolred (Kang et al., 2015).

The tropical Chinese hibiscus has been a subject of intensive hybridization for several centuries (Lawton, 2004; Thomson and Braglia, 2019) and currently thousands of cultivars are offered with a wide range of flower color and color combinations, and shapes (Singh and Khoshoo, 1970). Most of the Chinese hibiscus cultivars are multiple interspecific hybrids derived from the botanical species *Hibiscus kokio* Hillebr. ex Wawra, *Hibiscus arnottianus* Gray, *Hibiscus waimeae* A. Heller (2*x* = 2*n* = 80) and *Hibiscus schizopetalus* (Dyer) Hook. f. along with other *Hibiscus* species (Palmer and Palmer, 1954). Interestingly, the botanical species (a wild form) of *H. rosa-sinensis* has not been yet located (Shu, 2007). Linnaeus (1753) described the type specimen of *H. rosa-sinensis* in Sri Lanka. Thomson and Braglia (2019) speculated that Chinese hibiscus originated from South-East Asia and was spread over Pacific Islands by Polynesians. This shrub is adapted to tropical and subtropical regions; thus, its use as an outdoor ornamental plant in temperate regions is limited only to summer months. Exposure to temperatures lower than 11 °C reduces growth rate and causes severe leaf damage and eventually plant death (Karlsson et al., 1991). An improvement of the Chinese hibiscus for increased cold tolerance by conventional breeding methods may be unlikely because of the absence of genetic components regulating cold hardiness in this species. Creating somatic hybrids (fusant calluses) between transgenic *H. rosa-sinensis* and transgenic *Lavatera thuringiaca* L. (a cold-tolerant species) resulted in a significant improvement for chilling tolerance at conditions lethal to *H. rosa-sinensis*, although frost tolerance was not expressed (Vazquez-Thello et al., 1996). Unfortunately, plant regeneration from those fusant interspecific calluses was unsuccessful. Mercuri et al. (2010) developed a number of *H. rosa-sinensis* cultivars suitable for pot plant production and selected for the Mediterranean climate. These cultivars cannot be considered frost-resistant, but they have less pronounced tropical traits than their tropical parents.

One of the first successful attempts to create interspecific winter-hardy hibiscus hybrids belonging to the section *Muenchhusia* resulted in an amphidiploid hybrid named “Ai-Fuyo” (*H. muta-moscheutos* Kuwada), where *H. mutabilis* (2*x* = 2*n* = 92) was used as maternal parent and *H. moscheutos* (2*x* = 2*n* = 38) was the pollen donor (Kuwada, 1964). The F_1_ plants obtained from this hybridization were completely sterile. The doubling of the chromosome number was done in the F_1_ plants using colchicine, resulting in highly fertile amphidiploid (2*x* = 2*n* = 130) plants in the F_2_ generation. The amphidiploid hybrids expressed stem and leaf characteristics similar to these in *H. mutabilis*, but with larger flowers and more intense pink color. The plant growth habit was similar to that of *H. moscheutos*. This first known interspecific hibiscus hybrid has not been commercialized. Recently, Pounders and Sakhanokho (2016) successfully hybridized *H. mutabilis* (maternal plant) with *H. moscheutos* (pollen donor) and selected three interspecific cultivars resulting from that cross, i.e., “Hapa White”, “Hapa Red”, and “Hapa Pink”. Cold hardiness of these cultivars was inherited from the maternal component, the confederate rose (USDA Cold Hardiness Zones 7–9). When compared with the confederate rose, blooming period of the hybrids was extended from June through September. This extended flowering time was due in part to the hybrids being sterile. Similar to morphological traits reported by Kuwada (1964), these three hybrid cultivars were intermediate in growth habit and had increased basal branching, flower numbers and flower quality as compared with the parental species.

The attempts to create interspecific hybrids between the winter-hardy herbaceous *H. moscheutos* or woody *H. syriacus* and the tropical *H. rosa-chinensis* to introduce cold-tolerance to the latter species have not been successful (Yu et al., 1976; Paek et al., 1989; Kuligowska et al., 2013). Besides chromosomal incompatibility, other pre- and post-fertilization barriers prevented formation of embryos (Kuligowska et al., 2013). Kuligowska et al. (2013) examined pistils obtained from interspecific crosses between *H. rosa-sinensis* and *H. syriacus*, and between *H. rosa-sinensis* and *H. moscheutos* and found the occurrence of incompatibility in all cross combinations. Although pollination of *H. rosa-sinensis* with pollen of *H. syriacus* or *H. moscheutos* resulted in numerous pollen tubes penetrating the initial part of the pistil, only single pollen tubes were able to reach base of the style. The authors concluded that the lack of seed development, despite the ability of some pollen tubes to reach the ovule, indicated the occurrence of post-fertilization barriers in interspecific hybridization between tropical and winter-hardy hibiscus species. Interestingly, their subsequent studies (Kuligowska et al., 2016) revealed both pre- and post-fertilization hybridization barriers in crosses among *Hibiscus* species in the *Muenchhusia* section, e.g., *H. coccineus*, *Hibiscus laevis* and *H. moscheutos*. Pre-fertilization barriers included inhibition of pollen tube growth in some parents, although it did not cause a complete reproductive isolation between the parents. Post-fertilization barriers were expressed as seedling unviability, chlorosis, necrosis, stunted growth and albinism, and they were dependent upon specific parental plants.

In the recent years, a few herbaceous winter-hardy hibiscus cultivars have been released by Flemings Flower Fields, Lincoln, NE who claim they have created true hybrids between *H. moscheutos* or *H. coccineus* and the tropical *H. rosa-sinensis*, e.g., “Angelique” PP13,734 (United States Plant Patent, 2003a), “Pink Comet” PP13,751 (United States Plant Patent, 2003c), “Cherub” PP16,669 (United States Plant Patent, 2006), and “Satellite” PP23,759 (Justia Patents, 2012). “Angelique” is a multiple hybrid between a proprietary *H. moscheutos* “Bright Eye” (not patented) and a proprietary, unnamed *H. rosa-sinensis* hybrid (not patented). The cultivars Cherub and Pink Comet resulted from multiple crossings on a proprietary, unnamed *H. moscheutos* hybrid (not patented) with a proprietary, unnamed *H. rosa-sinensis* hybrid (not patented), although it is not stated in the patent applications if the parents were the same for both cultivars. “Satellite” is a multiple hybrid between a proprietary, unnamed interspecific *H. moscheutos* x *H. coccineus* hybrid (not patented) and a proprietary “TH-56” hybrid between *H. moscheutos* and *H. rosa sinensis* (not patented). The patent applications do not state what scientific methods were employed to verify the genetic nature of those hibiscus hybrid cultivars. We grew “Angelique” and “Cherub” in our evaluation at Vernon, TX during 2011–2013. Both cultivars easily hybridized (as maternal plants or pollen donors) with all winter-hardy hibiscus species in the section *Muenchhusia*; however, they did not hybridize (as maternal plants or pollen donors) with a number of *H. rosa-sinensis* cultivars in our concurrent tropical hibiscus breeding program. Therefore, the true hybrid nature of these interspecific hibiscus cultivars has yet to be scientifically verified.

## Flower Phenotypic Similarities Between Winter-Hardy and Tropical *Hibiscus* Hybrids

The winter-hardy hibiscus breeding program at Texas A&M AgriLife Research and Extension Center at Vernon was initiated in 2010 with the goal to develop cultivars with novel flower and foliage color and shape (Ledbetter, 2015). The inspiration for our breeding program were based on ideas of the two most progressive American amateur winter-hardy hibiscus breeders, C.S. Kennedy from Dublin, OH and Georgia Bost from Waller, TX. Kennedy, who started hybridizing North American herbaceous winter-hardy hibiscuses in the early 1950’s, wrote: *“I am convinced that our northern hibiscus has most of the potentials possessed by the tropicals. The search for these hidden traits, and the same could be said for any flower, will probably bring to the searcher more unalloyed happiness than he could find elsewhere”* (Kennedy, 1960). Georgia Bost, a passionate gardener who collected numerous herbaceous winter-hardy hibiscus species and developed and patented several new winter-hardy hibiscus cultivars on her Hibiscus Hill Plantation at Waller, TX in the 1980’s through her death in 2012, believed that extensive hybridization among North American herbaceous winter-hardy hibiscus species would eventually result in appearance of new flower colors similar to flower colors developed in the tropical Chinese hibiscus in the past 400 years (Casey, 1997). To create a broad genetic variability, we have conducted controlled hybridizations among several rosemallow hibiscus species native to the USA, e.g., *H. coccineus*, *H. militaris*, and *H. dasycalyx*, with a few commercial cultivars of *H. moscheutos*. The resulting interspecific hybrids consist of a combined genetic material of these North American hibiscus species. In contrast to findings by Kuligowska et al. (2016), only very rarely we observed genetic incompatibility between parents belonging to different hibiscus species in the section *Muenchhusia* expressed as a lack of fruit development and/or seed production after pollination. Our breeding strategy is selection of hybrids with desirable traits resulting from controlled hybridization between parents with advantageous flower, foliage and/or plant growth characteristics. One of the methods we apply to induce genetic mutations in our hybrids is exposure of seed to ionizing (microwave) radiation (Shirley et al., 1992). To date (2019), about 20,000 winter-hardy hibiscus hybrids have been created and evaluated in our breeding program.

The breakthrough winter-hardy hibiscus hybrid developed in our program was “Blue Angel” (Malinowski et al., 2012a). It was the first hibiscus hybrid in the *Muenchhusia* section with purple-bluish flower color that has not been previously reported in any of the parent species. The chemical identity of the novel pigment is currently being analyzed. Subsequent breeding resulted in development of a number of experimental lines with improved intensity of the blue flower color and commercialization of the first three bluish-flowering cultivars by J. Berry Nursery of Grand Saline, TX in 2018, including “Bleu Brulee”, “Brandy Bleu”, and “Cordon Bleu” (Fannin, 2018). Other novel color developed in our breeding program include silver, coral, magenta, fuchsia, purple, maroon, and numerous shades of red and pink colors as well as flowers with double and multiple color combinations. We have also developed the first chimera in winter-hardy hibiscus, released as “Robert Brown” in 2012 (Malinowski et al., 2012b). As our winter-hardy hibiscus hybrids have become genetically more and more complex, we have seen the first plants with flower colors, color combinations, and flower shapes phenotypically comparable with the tropical Chinese hibiscus, just like it was predicted by C.S. Kennedy and Georgia Bost. In this review, we compare flower phenotypic traits of these novel herbaceous winter-hardy hibiscus hybrids with that of their distant relatives, the tropical *H. rosa-sinensis* cultivars, and speculate the reasons for the resemblance. Most of the winter-hardy hibiscus hybrids described here are interspecific hybrids among four U.S.-native hibiscus species *H. coccineus*, *H. dasycalyx*, *H. militaris*, and *H. moscheutos*, except for the hybrids “14214-3”, “15375-2N”, and “15199-1^ST^” that are interspecific hybrids among *H. coccineus*, *H. militaris*, and *H. moscheutos*. We do acknowledge the numerous exceptional winter-hardy hibiscus cultivars developed by other breeders and commercial nurseries; however, those cultivars do not resemble tropical hibiscus in terms of flower phenotypic traits as do the hybrids developed in our breeding program. Thus, we do not include them in our review.

**Figure 1:**
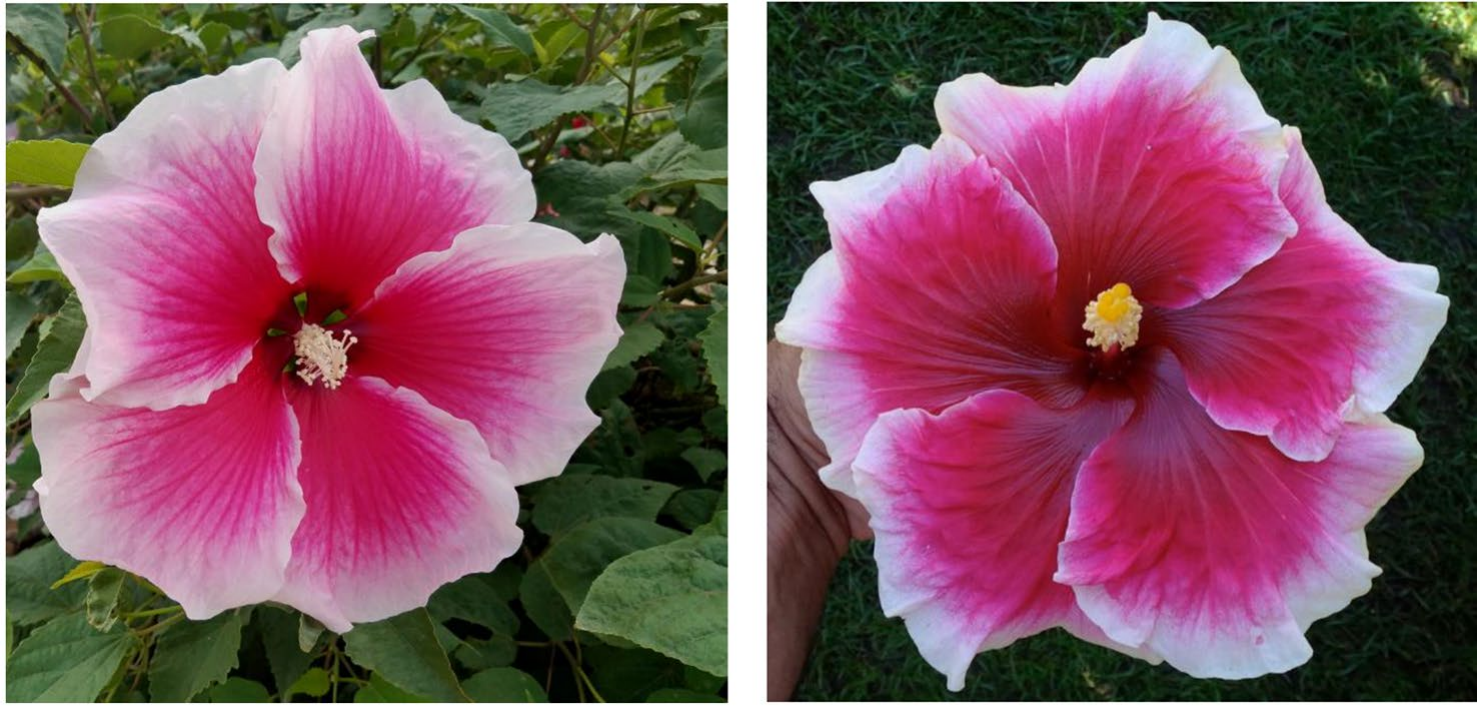
Flower color and shape of *H.* x *moscheutos* hybrid “TAMUS-5036” (left) and *H. rosa-sinensis* “Cindy’s Heart” (right). Photo credits: Dariusz P. Malinowski (“TAMUS-5036”) and Darren Eminian (“Cindy’s Heart”), Colorlicious Landscaping. Used with permission.

**Figure 2:**
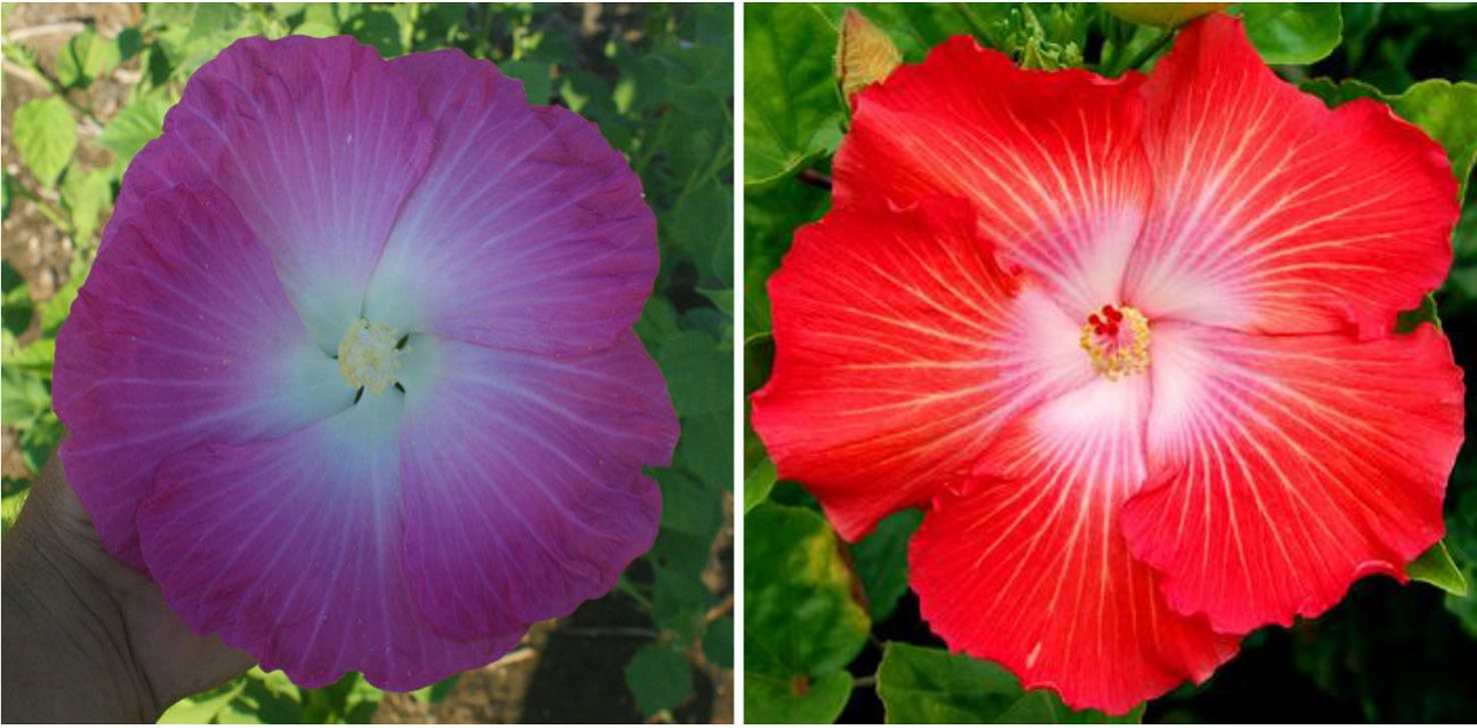
Flower color and shape of *H.* x *moscheutos* hybrid “TAMUS-4406” (left) and *H. rosa-sinensis* “Radiant” (right). Photo credits: Dariusz P. Malinowski (“TAMUS-4406”) and Cindy Black (“Radiant”), Hidden Valley Hibiscus. Used with permission.

To date, we have identified about 10 winter-hardy hibiscus hybrids with flowers resembling those of the modern cultivars of the tropical *H. rosa-sinensis*. Their similarities and differences in common phenotypic flower traits are presented in **Table 2**. Most of the winter-hardy hibiscus hybrids resulted from numerous hybridization cycles and multiple parents. Flowers of “TAMUS-5036” have a striking similarity in terms of the flower color and shape to the tropical cultivar “Cindy’s Heart” (**Figure 1**) released by Hidden Valley Hibiscus in 2008 (International Hibiscus Society, 2019). Another example are flowers of the winter-hardy hibiscus hybrid “TAMUS-4406” that closely resemble flowers of the tropical hibiscus cultivar “Radiant” (**Figure 2**).

**Table 2:** Summary of common and different phenotypical flower traits in winter-hardy hibiscus hybrids resembling tropical *H. rosa-sinensis* cultivars.

Winter-hardy hibiscus hybrid	Tropical hibiscus cultivar	Common phenotypical flower traits	Different phenotypical flower traits
“TAMUS-5036” Hybridization cycles: 8 Parental lines: 51	“Cindy’s Heart” Parents: self-pollination of “High Voltage” Hybridizer: Charles Black	Diameter: about 20 cm; Petals: overlapping with frilled edges; Petal color: magenta with white edges; Eye zone: marron; Veins: pink	Stigma: divided in five parts and white (“TAMUS-5036”) or uniform and yellow (“Cindy’s Heart”); Style: white (“TAMUS-5036”) or magenta (“Cindy’s Heart”)
“TAMUS-4406” Hybridization cycles: 7 Parental lines: 46	“Radiant” Parents: “Cosmic Gold” x “Saffron” Hybridizer: Charles Black	Diameter: about 20 cm; Petals: overlapping with slightly frilled edges; Eye zone: white, large, extending into white veins	Petal color: pink (“TAMUS-4406”) or red (“Radiant”); Stigma: white (“TAMUS-4456”) or red (“Radiant”)
“TAMUS-4614” Hybridization cycles: 6 Parental lines: 30	“Nightfire” Parents: “Honey Doo” x “Wedding Party” Hybridizer: Yasha and Daniel Brand	Diameter: about 16 cm; Petals: overlapping with slightly frilled edges; Petal color: maroon fading to blue; Eye zone: white, large, extending into white veins	Stigma: divided in five parts and white (“TAMUS-4614”) or uniform and maroon (“Nightfire”); Style: white (“TAMUS-4614”) or maroon (“Nightfire”)
“TAMUS-5075” Hybridization cycles: 9 Parental lines: 60	“Blue Me Away” Parents: Unknown Hybridizer: Sonny Stollings	Diameter: about 20 cm; Petals: overlapping, slightly wavy with frilled edges; Petal color: blue with light blue veins Stigma: divided in five parts; Anthers: yellow	Eye zone: bright red (“TAMUS-5075”) or dark purple (“Blue Me Away”); Style: light blue (“TAMUS-5075”) or light purple (“Blue Me Away”); Stigma: light blue (“TAMUS-5075”) or purple (“Blue Me Away”)
“15681-1GR” Hybridization cycles: 6 Parental lines: 27	“Chariots of Fire” Parents: “The Path” x “Ring of Fire”; Hybridizer: Charles Black	Diameter: about 16 cm; Petals: overlapping with slightly frilled edges; Petal color: reddish-orange with light pink to white veins; Eye zone: dark red; Stigma: reddish-orange	Stigma: divided in five parts (“15681-1GR”) or uniform (“Chariots of Fire”)
“TAMUS-4621” Hybridization cycles: 8 Parental lines: 22	“Hawaiian Dot” Parents: unknown; Hybridizer: Edward Teas	Diameter: about 18-20 cm; Petals: not overlapping, elongated; Petal color: white; Eye zone: dark red; Style: white and very long	Stigma: divided in five parts (“TAMUS-4621”) or uniform (“Hawaiian Dot”); Anthers: white (“TAMUS-4621”) or yellow (“Hawaiian Dot”)
“15375-2N” Hybridization cycles: 5 Parental lines: 12	“Ghost” Parents: unknown; Hybridizer: Gordon Shigeura	Diameter: about 16 cm; Petals: overlapping, textured; Petal color: white; Eye zone: not present; Style: white; Stigma: divided in five parts	Petals: frilled edges (“Ghost)
“16755-2N” Hybridization cycles: 8 Parental lines: 56	“161122-TR” Parents: *H. rosa-sinensis* unnamed seedling x *H. schizopetalus*; Hybridizer: Dariusz Malinowski	Diameter: about 15 cm; Petals: overlapping, ribbed, strongly curved to the back; Style: red and very long; Stigma: red, divided in five parts; Anthers: yellow	Petal color: reddish-crimson (“16755-2N”) or bright red (“161122-TR”); Eye zone: dark-red (“16755-2N”) or not present (“161122-TR”);
“15199-1ST” Hybridization cycles: 3 Parental lines: 6	Unnamed hybrid Parents: Unknown Hybridizer: George C. Charles Origin: Mauritius	Diameter: about 12 cm; Petals: not overlapping, narrow at the base and wider at the top, slightly ribbed; Eye zone: not present; Style: red; Stigma: red, divided in five parts; Anthers: yellow	Petal color: ruby-red (“15199-ST”) or red (Unnamed hybrid)
“TAMUS-5050” Hybridization cycles: 7 Parental lines: 43	“Moorea Abyss” Parents: “Moorea Brownship” x “Moorea Maie Blue” Hybridizer: Atiu Charles	Petals: strongly overlapping and curved. Style: white, divided in 5 parts;	Diameter: about 15 cm (“TAMUS-5050”) or 18-20 cm (“Moorea Abyss”); Petal color: pink with random small white dots (“TAMUS-5050”) or blue (“Moorea Abyss”); Eye zone: red (“TAMUS-5050”) or pale blue (“Moorea Abyss”); Stigma: white (“TAMUS-5050”) or orange (“Moorea Abyss”); Pollen: white (“TAMUS-5050”) or yellow (“Moorea Abyss”)

“Radiant” was released by Hidden Valley Hibiscus in 2011 (International Hibiscus Society, 2019).

One of the best achievements in our breeding program was “Blue Angel”, the first winter-hardy hibiscus hybrid with a bluish flower color (Malinowski et al., 2012a). Rakhimkhanov et al., (1970;^1973^) were the first researchers looking for the illusive blue pigment in winter-hardy hibiscus. They hybridized the north American species of *H. moscheutos*, *H. militaris*, and *H. coccineus*. Although the authors did not create a blue-flowering hibiscus hybrid, they isolated two novel anthocyanin glycosides from their hybrid hibiscus flowers, gossypicyanin, and chrysanthemin. Another anthocyanin pigment, delphinidin, was found in some of the BOSTx^®^HHHybrid Pink series of winter-hardy hibiscus hybrids developed by Georgia Bost (Puckhaber et al., 2002), but none of their hybrids had a blue flower color. Anthocyanins are responsible for blue, purple, red, and pink flower colors, depending on the chemical structure of individual pigment molecules and the pH the pigment molecules are exposed to (Zhao and Tao, 2015). In contrast to herbaceous winter-hardy hibiscus species of the section *Muenchhusia*, blue flower color has been achieved in the woody winter-hardy *H. syriacus* (“Bluebird”, “Blue Chiffon™”, “Azurri Blue Satin^®^”), and the tropical *H. rosa-sinensis* (numerous cultivars). Blue pigment did not exist in tropical hibiscus until the early 1950’s. The first mention of a *H. rosa-sinensis* hybrid with “a hint of lavender” was by Wilcox and Holt (1913) in Hawaii, but most likely the first light lavender-flowering cultivar was Hale Blue of unknown origin (Black, 2019b). Although the cultivar became extinct soon after it was discovered, it is speculated that “Hale Blue” might have been a botanical species because its progeny propagated from seeds was true to the parental plants. “Hale Blue” was a parent of the earliest known blue and brown-flowered varieties Myrna Loy, Dolores Del Rio, Mahogany, and Lavender Sky. Currently, there are numerous blue-flowering tropical hibiscus hybrids available commercially and in collections (International Hibiscus Society, 2019).

**Figure 3:**
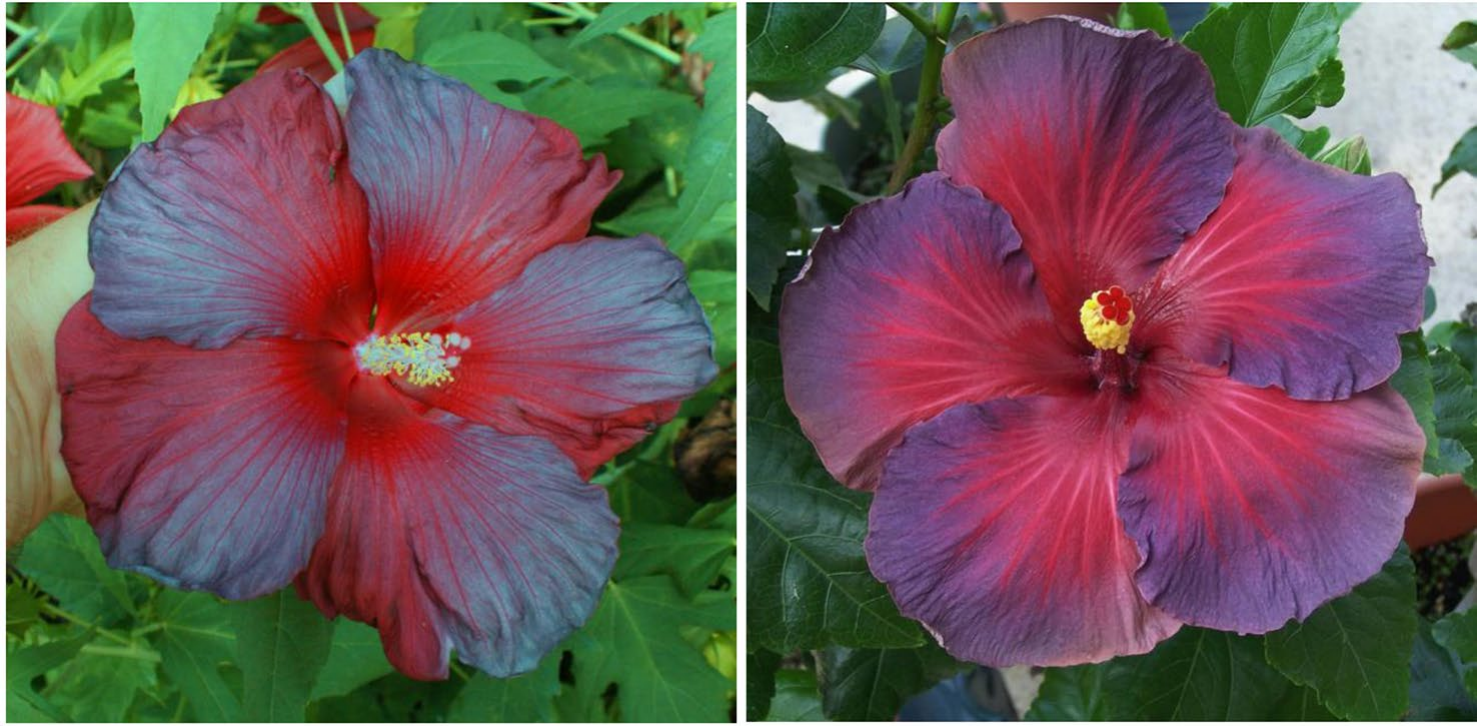
Flower color of *H.* x *moscheutos* hybrid “TAMUS-4614” (left) and *H. rosa-sinensis* “Nightfire” (right). Photo credits: Dariusz P. Malinowski (“TAMUS-4614”) and Naralin Valanna (“Nightfire”), https://i.pinimg.com/originals/45/49/4b/45494bf39000d95df552c27d01049017.jpg.

**Figure 4:**
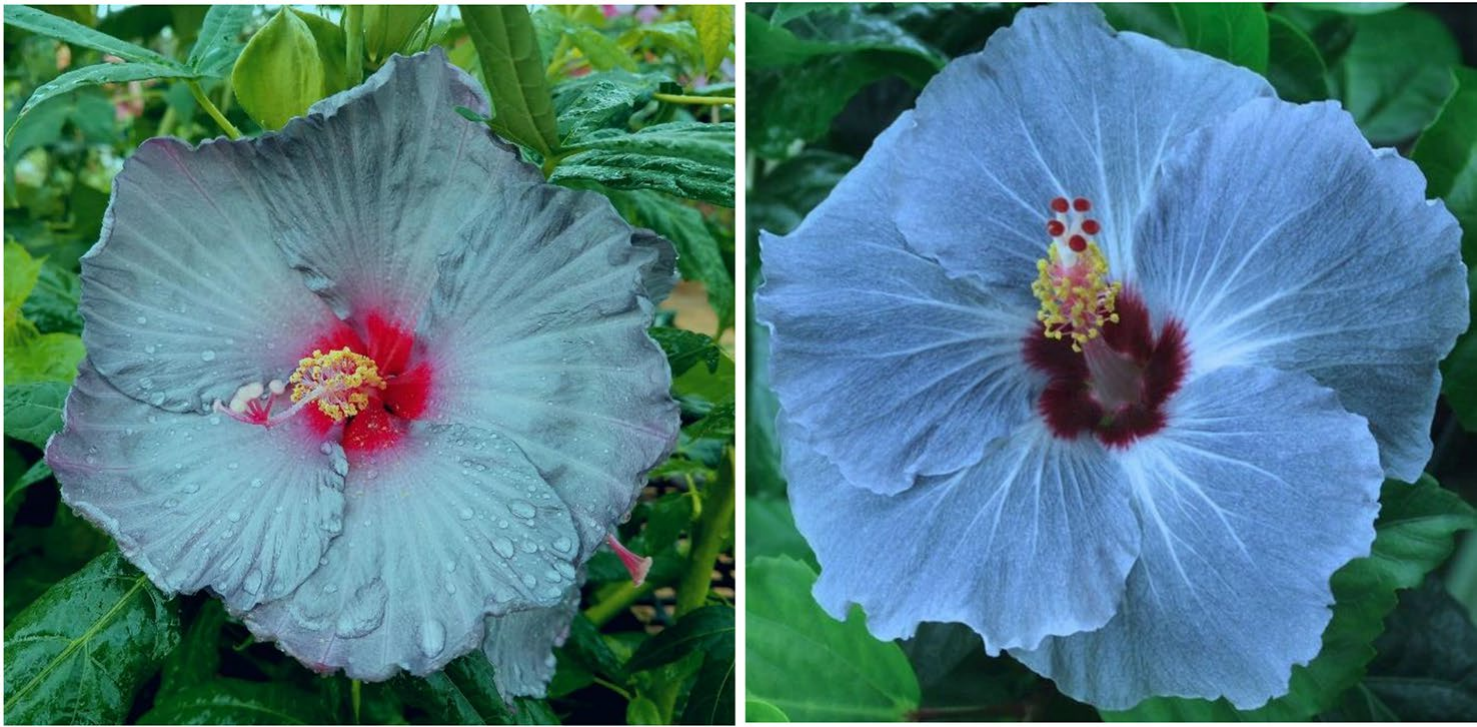
Flower color of *H.* x *moscheutos* hybrid “TAMUS-5075” (180865 GR) (left) and *H. rosa-sinensis* “Blue Me Away” (right). Photo credits: Dariusz P. Malinowski (“TAMUS-5075”) and David Thompson (“Blue Me Away”). Used with permission.

In a selective breeding process, we were able to combine both the bluish flower color of “Blue Angel” and maroon flower color of our winter-hardy hibiscus hybrid “TAMUS-3620” in one flower. The resulting hybrid “TAMUS-4614” resembles closely the tropical hibiscus cv. Nightfire (**Figure 3**). Another descendant of “Blue Angel is the hybrid “TAMUS-5075” with blue flowers resembling the tropical cultivar “Blue Me Away” (**Figure 4**). We improved “TAMUS-5075” for a more intense blue flower color and a compact plant growth when compared with “Blue Angel”. Flowers of “TAMUS-5075” may occasionally show traces of lavender tint, especially in colder temperatures. Future research should reveal the chemical nature of the novel blue pigment in the blue-flowering winter-hardy hibiscus hybrid series developed in our program and its similarity to the blue pigment present in tropical hibiscus hybrids.

**Figure 5:**
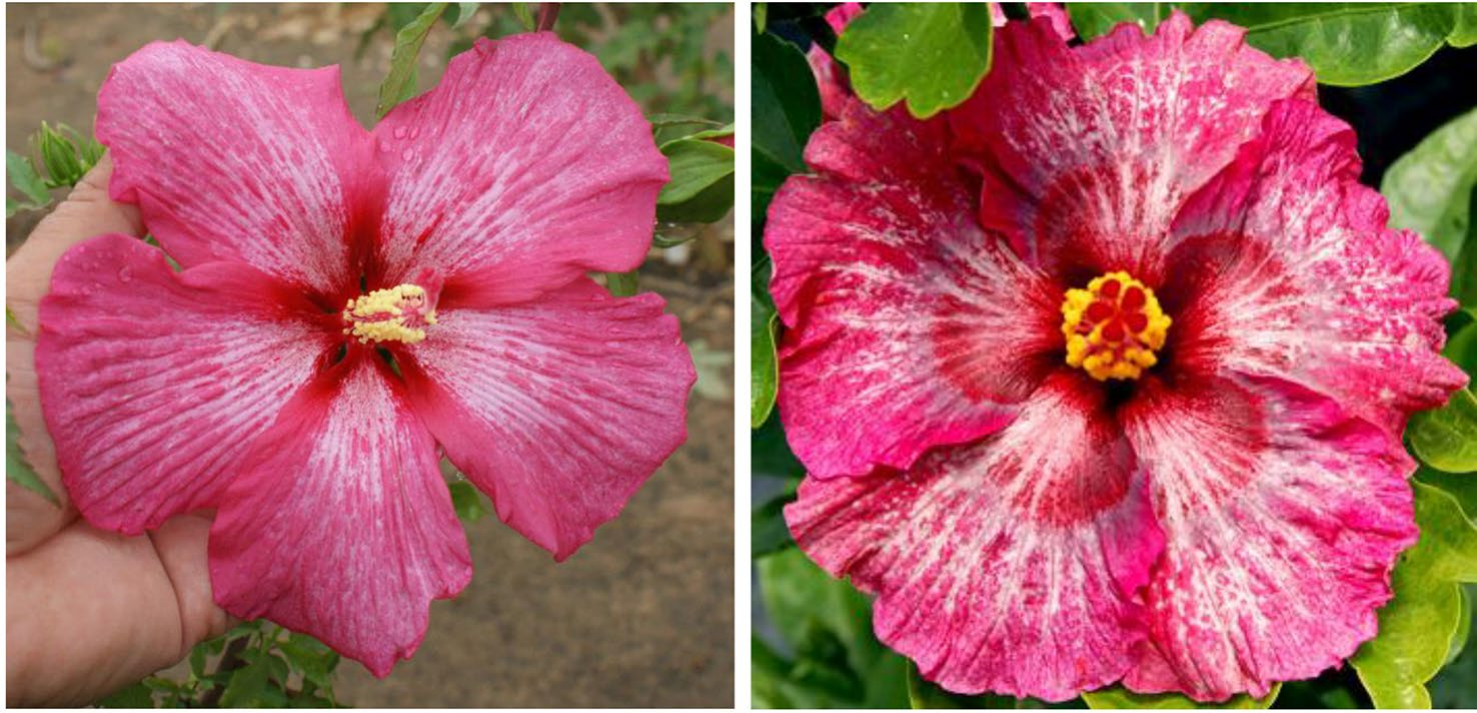
Flower color and shape of *H.* x *moscheutos* hybrid “14214-3” (left) and *H. rosa-sinensis* “Glitz ‘n’ Glitter” (right). Photo credits: Dariusz P. Malinowski (“14214-3”) and Cindy Black (“Glitz ‘n’ Glitter”), Hidden Valley Hibiscus. Used with permission.

**Figure 6:**
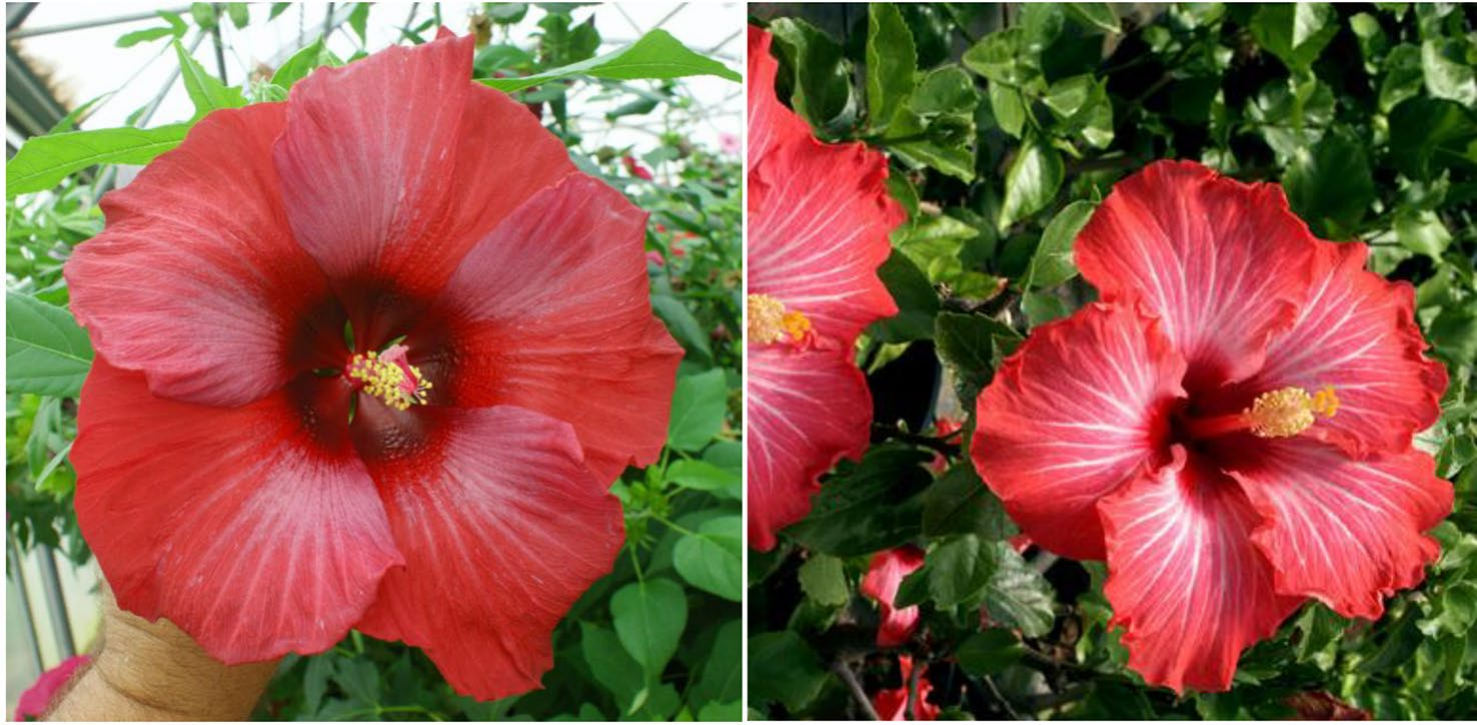
Flower color and shape of *H.* x *moscheutos* hybrid “15681-1GR” (left) and *H. rosa-sinensis* “Chariots of Fire” (right). Photo credits: Dariusz P. Malinowski (“15681-1GR”) and Cindy Black (“Chariots of Fire”), Hidden Valley Hibiscus. Used with permission.

The winter-hardy hibiscus “14214-3” was one of the first hybrids in our breeding program with a “zebra” or “batik”-type coloration of flowers. Such a coloration has been known in a few tropical hibiscus hybrids. “14214-3” closely resembles the tropical hibiscus “Glitz ‘n’ Glitter” (**Figure 5**). Another example of such flower coloration is the hybrid “15681-1GR” with flowers closely resembling the tropical hibiscus “Chariots of Fire” (**Figure 6**).

**Figure 7:**
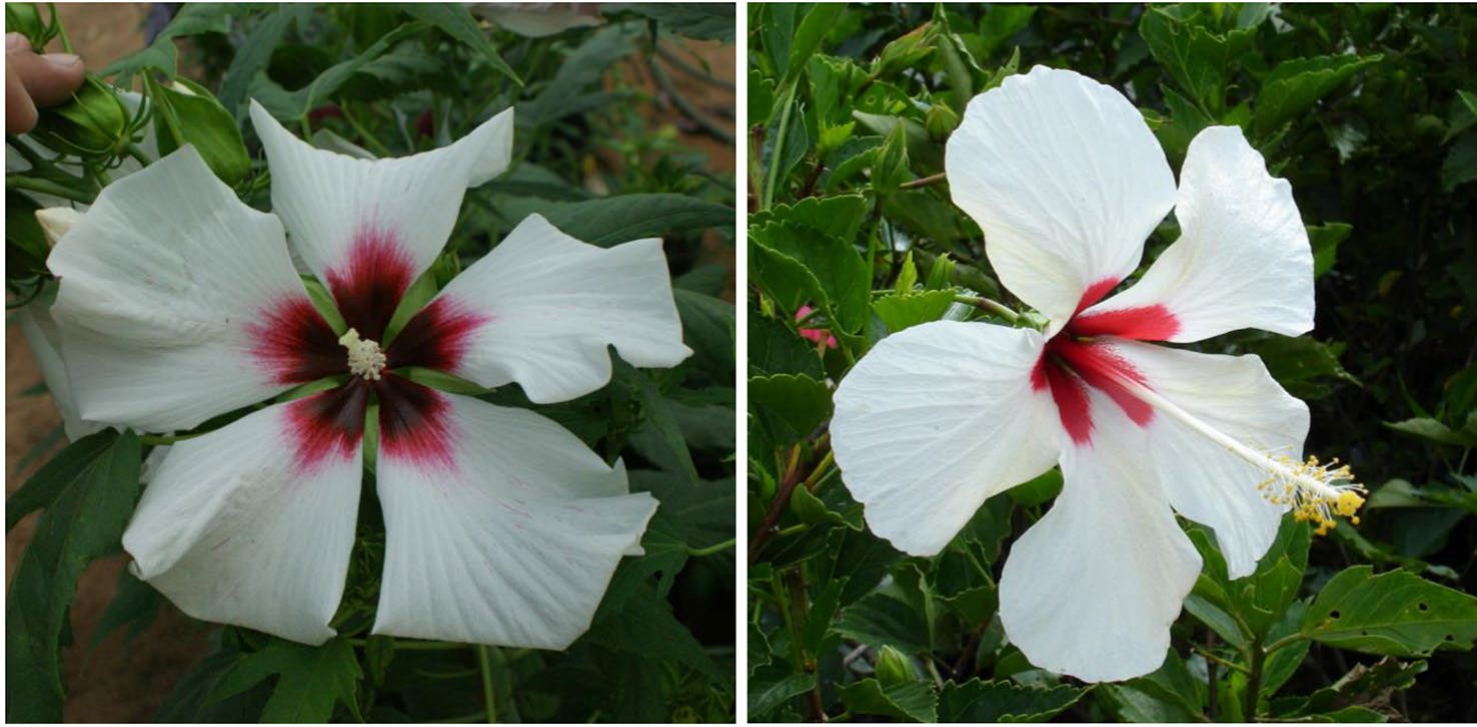
Flower color and shape of *H.* x *moscheutos* hybrid “TAMUS-4621” (left) and *H. rosa-sinensis* “Hawaiian Dot” (right). Photo credits: Dariusz P. Malinowski (“TAMUS-4621”) and Georgi Stanev (“Hawaiian Dot”). Used with permission.

The winter-hardy hibiscus hybrid “TAMUS-4621” is an example of hibiscus with windmill-shape flowers. “TAMUS-4621” closely resembles the tropical hibiscus cultivar “Hawaiian Dot” (**Figure 7**). The cultivar “Hawaiian Dot” is also known under the names “Melba” and “White Wings” (International Hibiscus Society, 2019). Flowers of both cultivars have a very tropical look.

The lack of an eye zone and pure white flower traits in winter-hardy hibiscus are inherited from the *H. coccineus* f. *alba* natural mutant, in which it is controlled by a single diallelic “white flower” locus with red flower color (W) being completely dominant to white (w) (Gettys, 2012). Thus, a white flowering hibiscus hybrid would be a recessive homozygote (ww) in that allele. We selected a few hybrids with pure white flowers in our breeding program. One of them is “15375-2N”. Its pollen parent, the hybrid “10250-3”, originated from a self-pollination of a breeding line “HM-2007-RaspP” that had in its parentage the cultivar “Raspberry Rose”. “Raspberry Rose” originated from a red-flowering *H. coccineus*, that might have been heterozygotic in the Ww allele. The hybrid “15375-2N” may be a homozygote in the ww allele because its maternal parent “10250-3” also originated from “Raspberry Rose”. However, despite lacking an eye zone, the flower color of “15375-2” is not pure white, it has a subtle pink tint. Gettys (2012) concluded that the biochemistry of the pure white flower color in *H. coccineus f. alba* was unknown.

**Figure 8:**
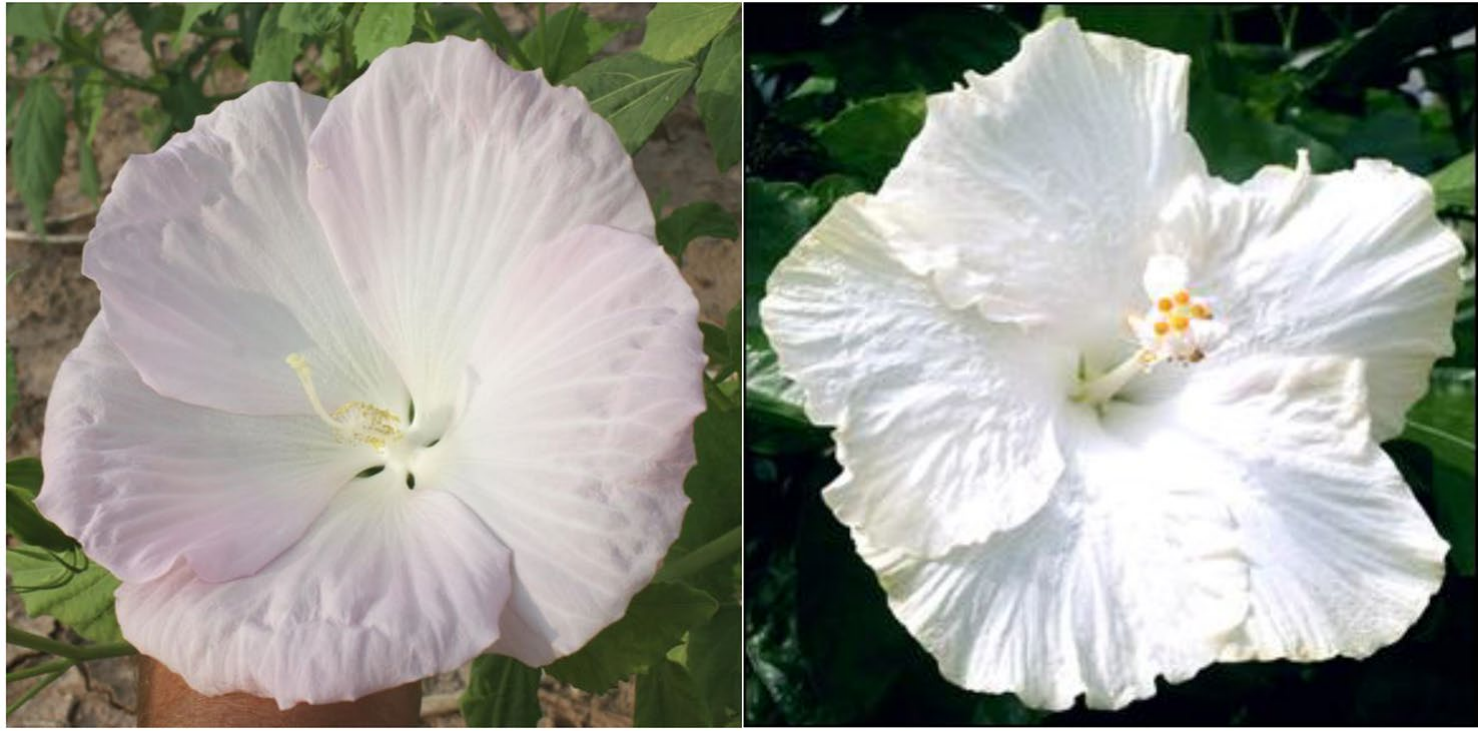
Flower color and shape of *H.* x *moscheutos* hybrid “15375-2N” (left) and *H. rosa-sinensis* “Ghost” (right). Photo credits: Dariusz P. Malinowski (“15375-2N”) and Cindy Black (“Ghost”), Hidden Valley Hibiscus. Used with permission.

Flowers of “15375-2” resemble those of the tropical hibiscus “Ghost”, especially regarding their shapes (**Figure 8**). There are about 300 registered cultivars of white-flowering tropical hibiscus hybrids (International Hibiscus Society, 2019), while there are only a few commercial cultivars of pure white-flowering winter-hardy hibiscus, i.e., Blue River, Blue River II, and Snow White (also known as cv. Swamp Angel). All of them inherited the flower shape from either *H. coccineus* or *H. moscheutos* and differ in this trait from the hybrid “15375-2” or tropical hibiscus flowers.

**Figure 9:**
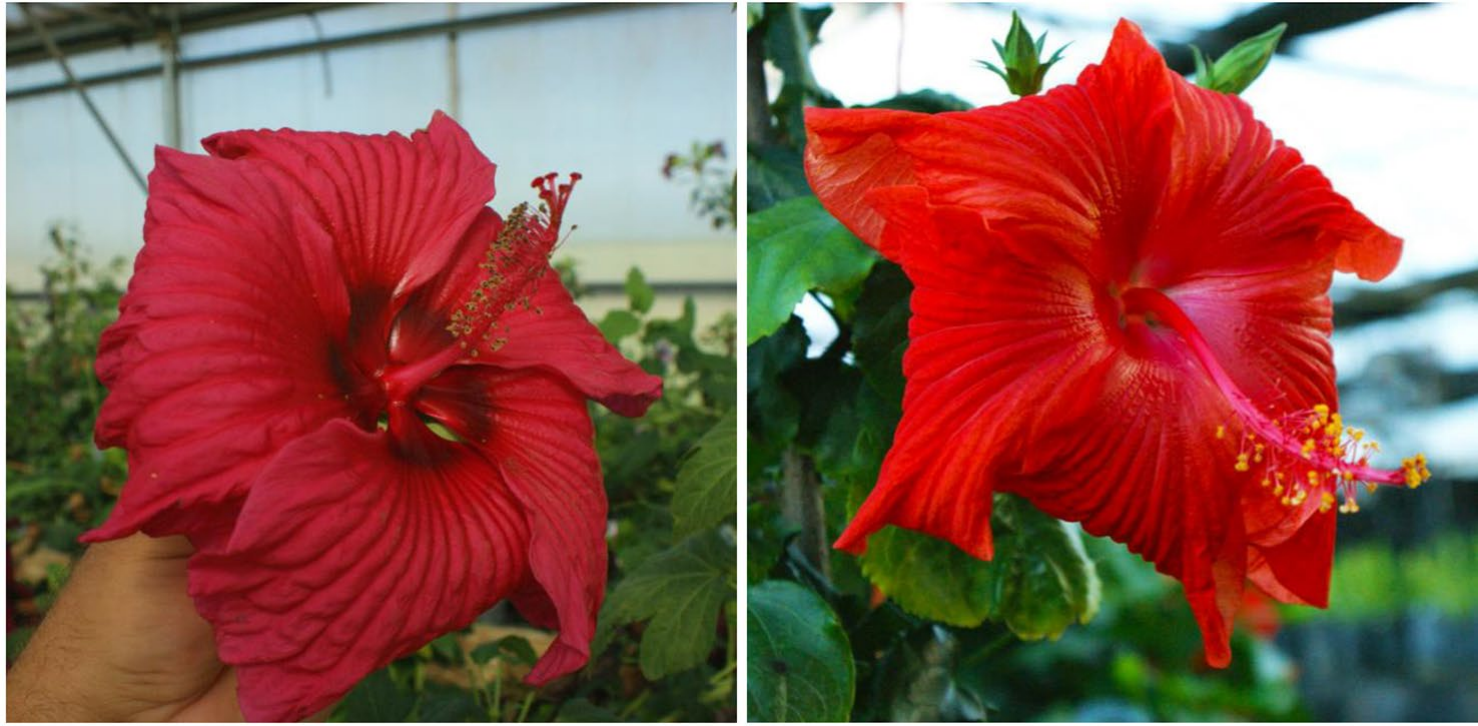
Flower color and shape of *H.* x *moscheutos* hybrid “16755-2N” (left) and an interspecific tropical hybrid *H. rosa-sinensis* x *H. schizopetalus* “161122-TR” (right). Photo credit: Dariusz P. Malinowski.

The winter-hardy hibiscus hybrid “16755-2N” has a novel flower shape never seen before in the *Hibiscus* section *Muenchhusia*. Petals have distinct ribs and are strongly curved backwards. The flowers closely resemble those of a tropical hibiscus “161122-TR” (**Figure 9**), an interspecific hybrid (F_1_) between an unnamed *H. rosa-sinensis* and *H. schizopetalus* selected by D.P. Malinowski in 2016.

**Figure 10:**
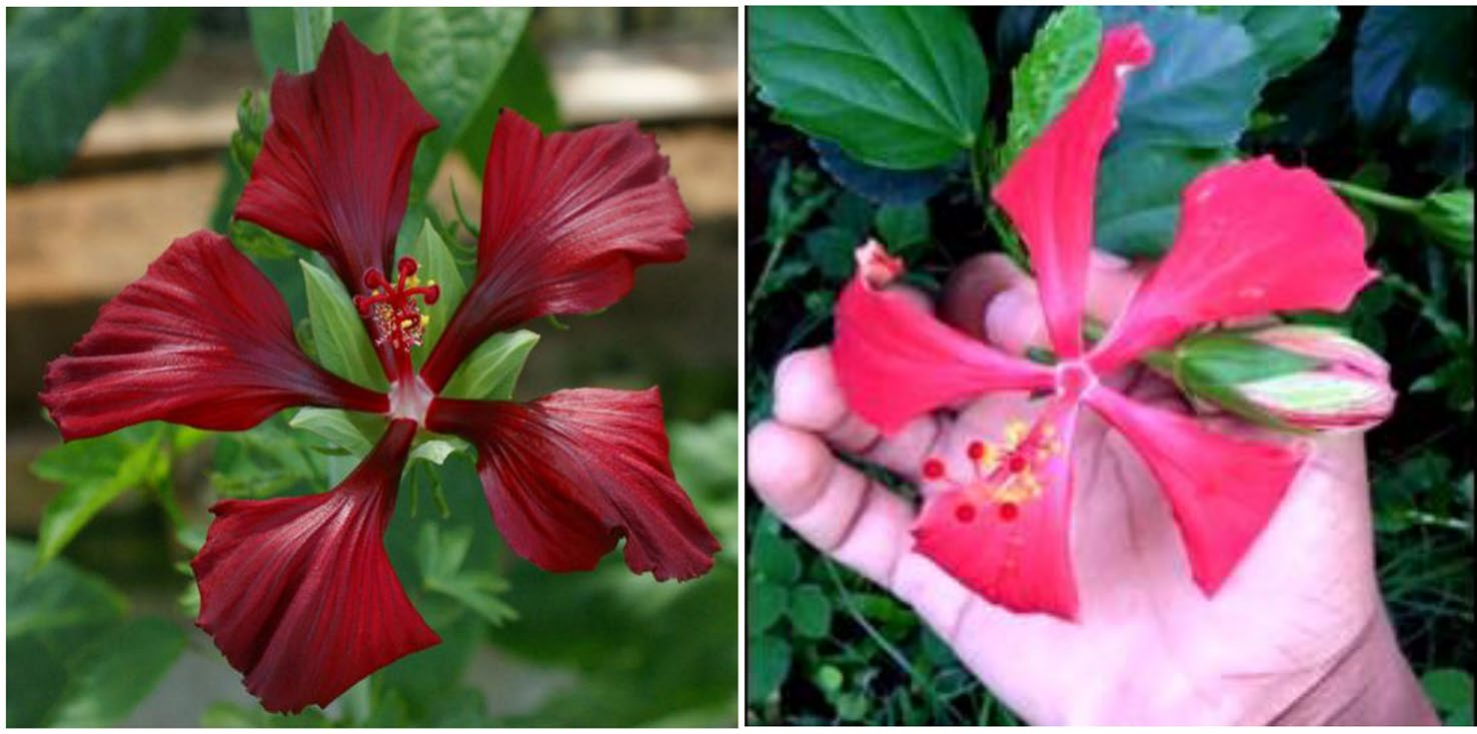
Flower color and shape of *H.* x *moscheutos* hybrid “15199 ST” (left) and an unnamed *H. rosa-sinensis* hybrid (right). Photo credits: Dariusz P. Malinowski (15199 ST”) and George C. Charles (*H. rosa-sinensis* hybrid), Mauritius. Used with permission.

The winter-hardy hibiscus hybrid “15199 ST” has also a new flower shape resembling that of an unnamed tropical hibiscus hybrid discovered by George C. Charles in Mauritius (**Figure 10**). Interestingly, even anthers are located at the upper part of the style, similar as for a tropical hibiscus flower.

## The Development of a “Double Flower” Phenotype of Winter-Hardy Hibiscus

**Figure 11:**
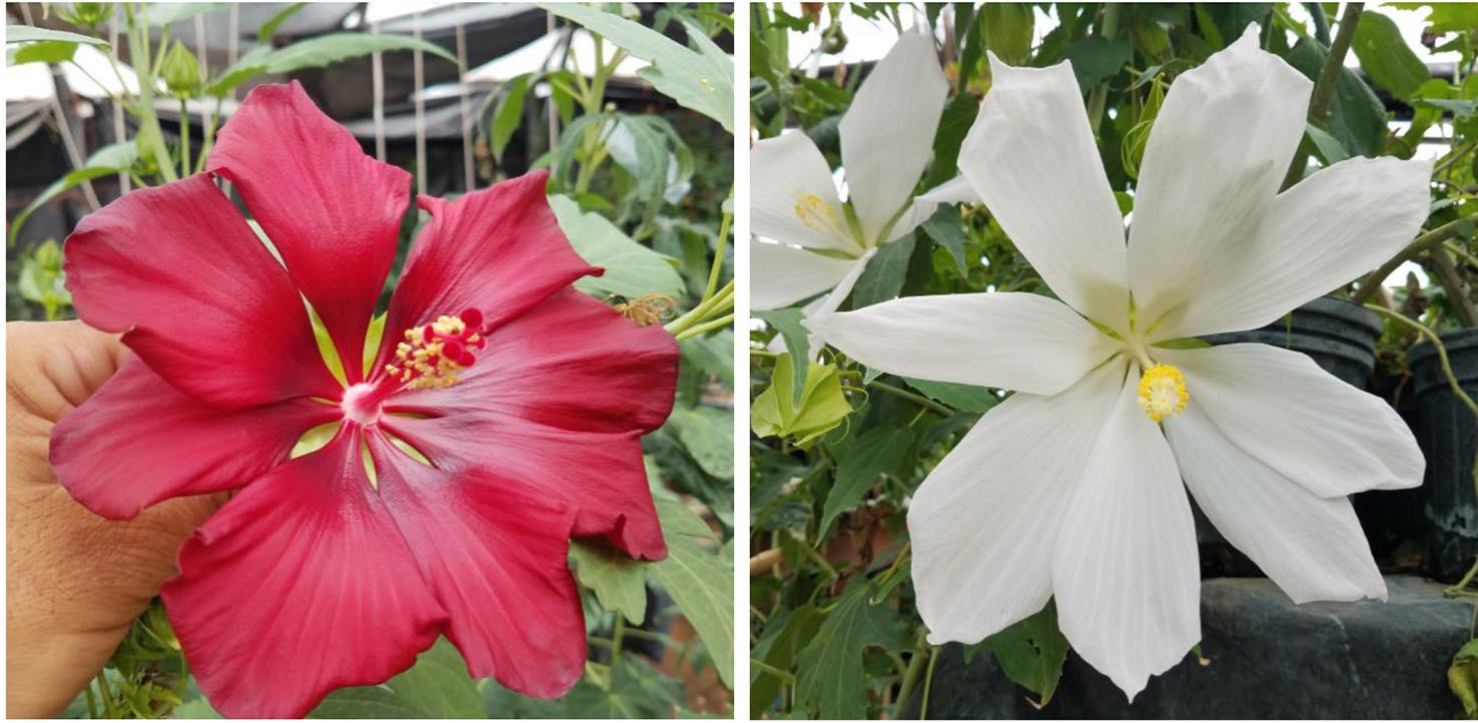
Examples of *H.* x *moscheutos* hybrids that tend to produce flowers with more than the regular five petals: “ST199-2” (left) and “16751 GR” (right). Photo credits: Dariusz P. Malinowski.

**Figure 12:**
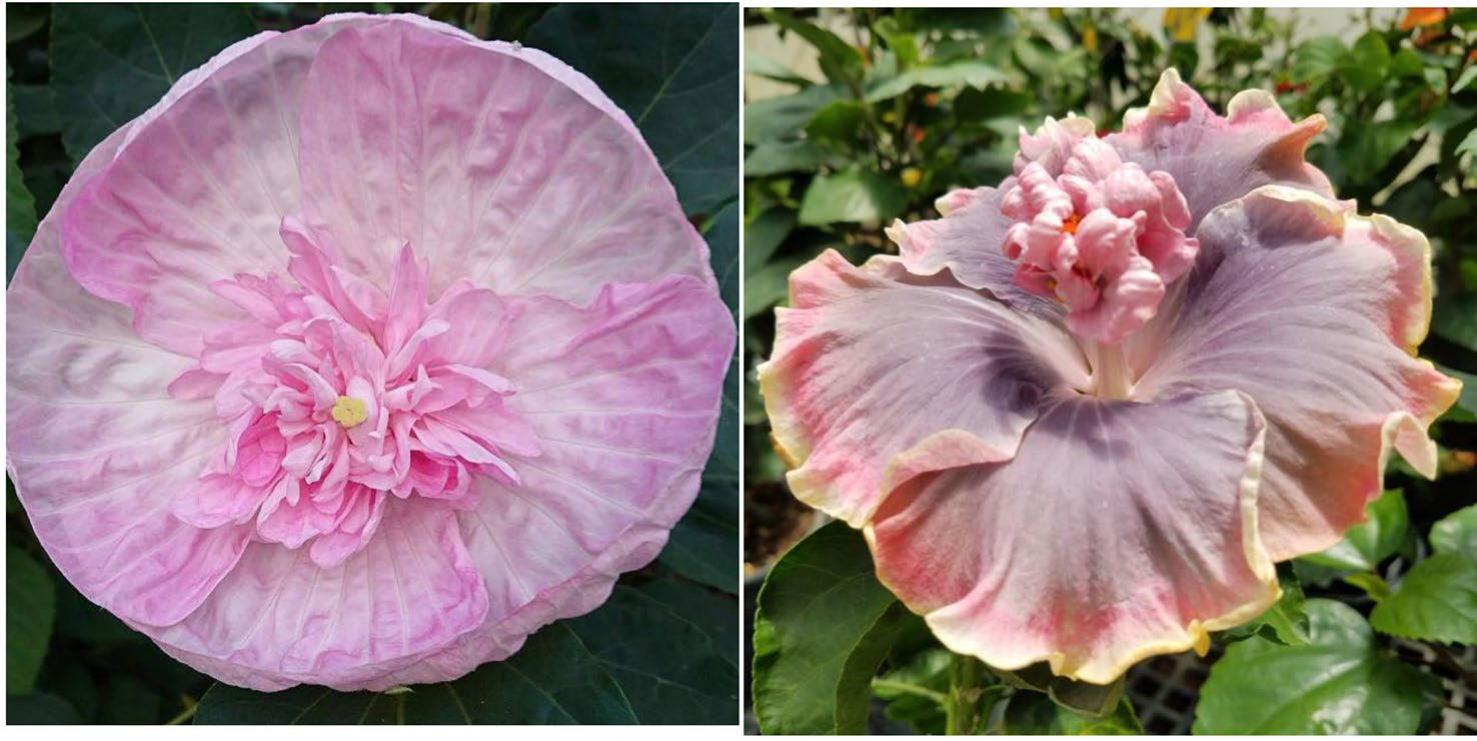
Flower shape of *H. moscheutos* “Serdzse Matery” (left) and *H. rosa-sinensis* “Moorea Boondah Boo” (right). Photo credits: Lyudmila Zhurenko (“Serdzse Matery”) and Dariusz P. Malinowski (“Moorea Boondah Boo”). Used with permission.

All herbaceous winter-hardy hibiscus species in the section *Muenchhusia* have flowers consisting of five petals. In the 1950’s, a *H. moscheutos* cultivar Annie J. Hemming was released that occasionally produced flowers with six or seven petals (Hemming, 1952; Hemming, 1959). Kennedy (1960) reported a hybrid with a similar trait in his breeding program, but there is no indication that a multi-petal cultivar has ever been developed from it. We also occasionally see herbaceous winter-hardy hibiscus hybrids with more than five petals in our evaluations; however, it seems to be a random phenomenon and the trait does not seem heritable. However, we have identified two related hybrids that often produce flowers with up to eight petals (**Figure 11**). These extra petals seem to be a result of sepal, not stamen modifications (Meyerowitz et al., 1989). Double-flower forms often arise when some or all the stamens in a flower are replaced by petals. These types of mutations, where one organ in a developing organism is replaced with another, are defined as homeotic mutations and are known in many plant families, including Malvaceae (Ronse De Craene, 2003). Although numerous examples of homeosis resulting in multi-petal flowers have been described in *H. rosa-sinensis* (MacIntyre and Lacroix, 1996; Salamah et al., 2018), only one such a hybrid is known in *H. moscheutos*, the cv. “Serdzse Matery” (Mother’s Heart) shown in **Figure 12**. “Serdzse Matery” was selected by Marina Mariny, an amateur hybridizer from Ukraine, in 2013 and introduced in Ukraine in 2015 (Zhurenko, 2019). Although the additional petals are not fully developed in “Serdzse Matery”, they clearly originate from stamens, just like it has been observed in some tropical hibiscus hybrids with “full” (*flore pleno*) flowers. At this early stage of a multi-petal flower formation, flowers of the winter-hardy hibiscus “Serdzse Matery” closely resemble flowers of *H. rosa-sinensis* “Moorea Boondah Boo” and similar tropical hybrids with this type of homeotic mutations. “Moorea Boondah Boo” was hybridized by Atiu Charles in French Polynesia in 2009, using “Moorea Abyss” and “Moorea Imperial Bloosom” as parents (International Hibiscus Society, 2019).

**Figure 13:**
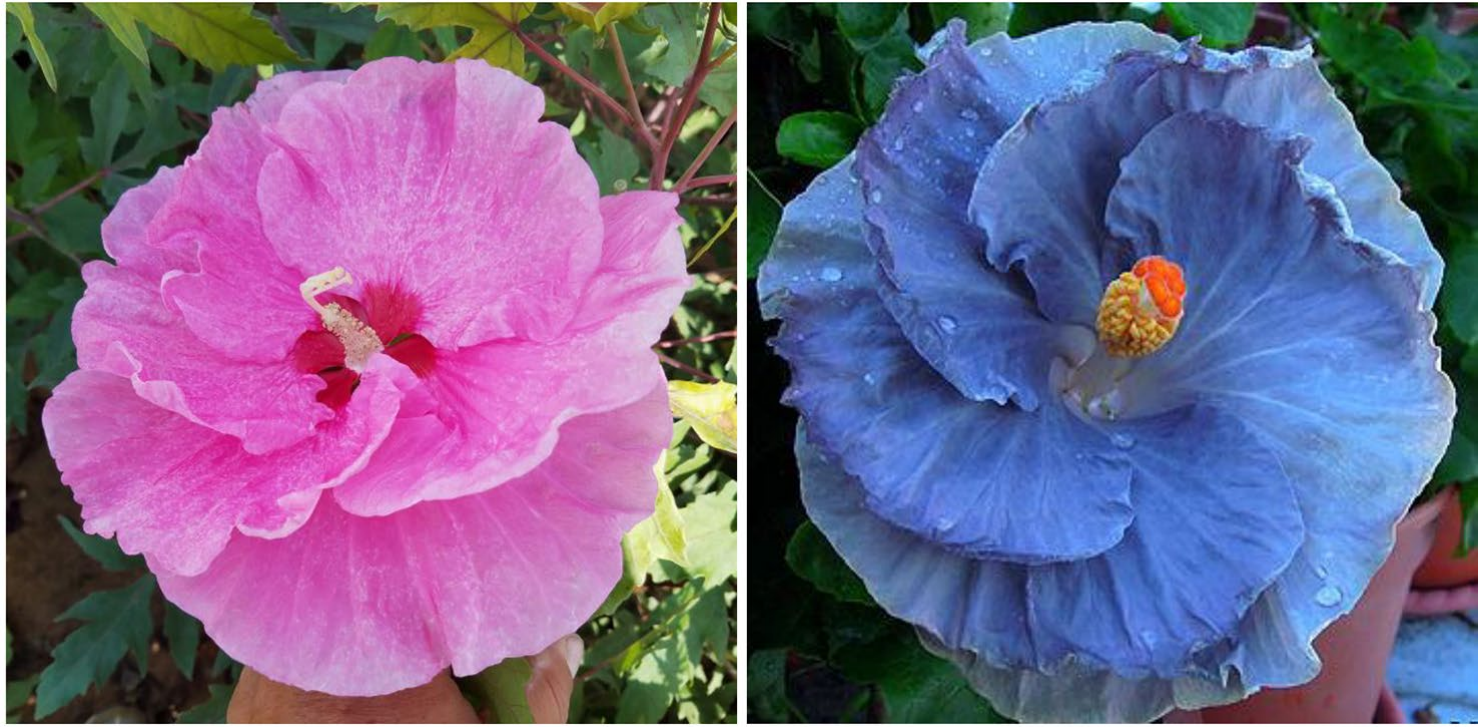
Flower shape of *H.* x *moscheutos* hybrid “TAMUS-5050” (left) and *H. rosa-sinensis* “Moorea Abyss” (right). Photo credits: Dariusz P. Malinowski (“TAMUS-5050”) and Linda Lee (“Moorea Abyss”), http://www.prettilife.com/hibiscus.

One approach to mimic double flowers in herbaceous winter-hardy hibiscus hybrids is to create hybrids with flowers where petals are shaped in a way that creates an illusion of a full, double flower. The most dramatic example is the hybrid “TAMUS-5050” resembling flower structure of the tropical hibiscus “Moorea Abyss” (**Table 2** and **Figure 13**). “Moorea Abyss” was hybridized by Atiu Charles in French Polynesia and registered in 2009 (International Hibiscus Society, 2019).

## Winter-Hardy Hibiscus Hybrids With Yellow and Orange Flowers

Yellow flower color, in addition to blue color, has been a hybridizers” goal since the beginning of systematic breeding efforts of herbaceous winter-hardy rosemallows (Winters, 1970). Flavonoids, particularly anthocyanidin glycosides, are the major flower pigments and are responsible for most of the flower colors (Martin and Gerats, 1993). Flavonoids are also present in herbaceous and woody winter-hardy hibiscus species (Puckhaber et al., 2002). The anthocyanin pigments are responsible for all shades of blue, lavender, purple, and red colors (Zhao and Tao, 2015). A group of flavonoids, the flavonol compounds and particularly quercetin, are also found in herbaceous winter-hardy hibiscus flowers (Puckhaber et al., 2002). Flavonol compounds are responsible for yellow flower color in the related genus, cotton (*Gossypium* spp.) (Tan et al., 2013). In tropical hibiscus, the yellow, orange, and red flower colors are regulated by carotenoids (Shobha et al., 1999); however, flavonols are also known to produce pale yellow flower colors in some tropical hibiscus hybrids, e.g., “Acadian Spring” (Black, 2019a).

Carotenoids have not been found in herbaceous winter-hardy hibiscus species in the section *Muenchhusia* (Puckhaber et al., 2002); thus, creating hybrids with yellow or orange flowers using traditional breeding methods may be a challenge. The cultivars “Old Yella” PP13630 and “New Old Yella” PP23698 developed by Flemings Flower Fields express light creamy to very light yellowish flower color that varies significantly depending on a range of environmental factors (United States Plant Patent, 2003b; United States Plant Patent, 2012), similarly as in the tropical hibiscus “Acadian Spring” (Black, 2019a). The chemistry of pigments involved in the flower color of these herbaceous winter-hardy hibiscus hybrids has yet to be determined.

**Figure 14:**
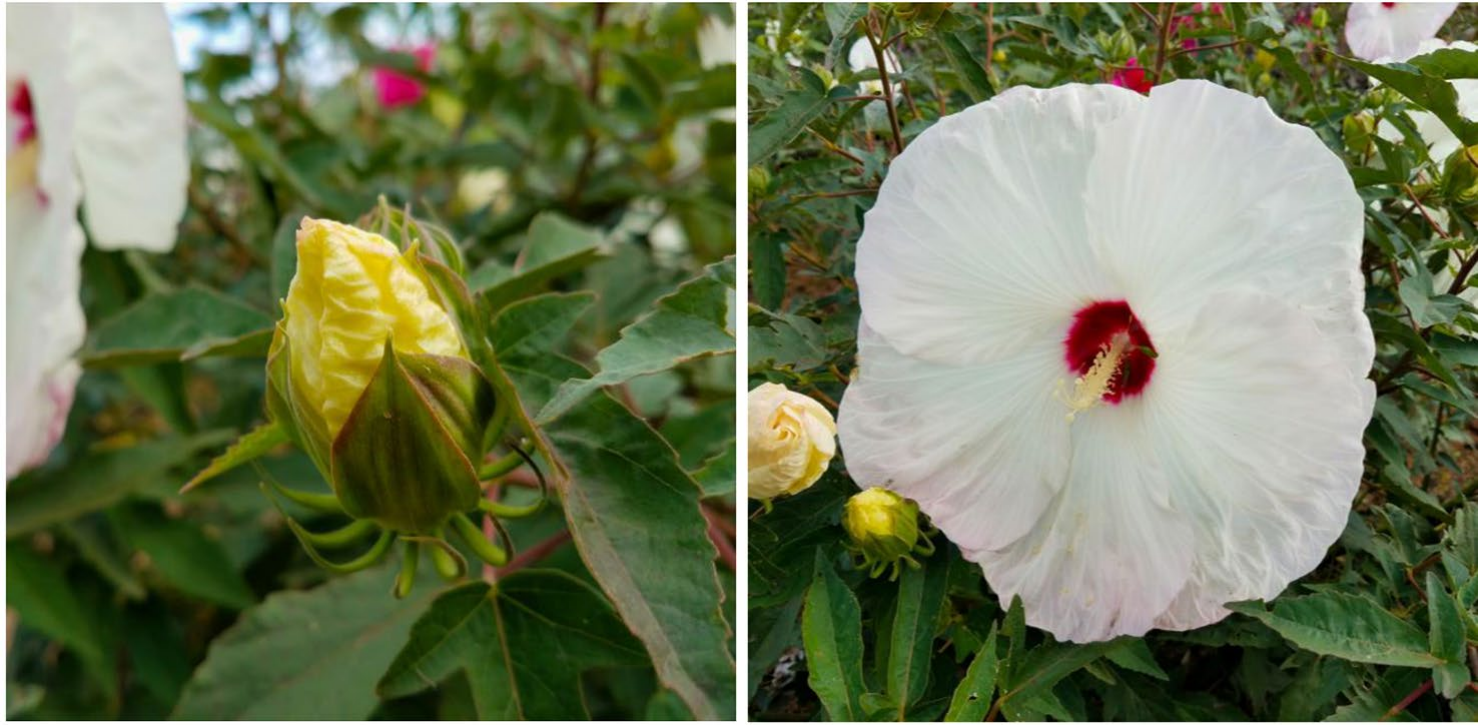
Opening flower bud (left) and fully opened flower color of *H.* x *moscheutos* hybrid “180074-M1W”. Photo credits: Dariusz P. Malinowski.

We have selected a herbaceous winter-hardy hibiscus hybrid “180007-M6W” which opening flower buds has yellow color, but the yellow pigment fades to creamy and finally white color as the flower weathers during the day (**Figure 14**). The winter-hardy hibiscus “180074-M1W” is a complex hybrid resulting from multiple hybridization cycles of several breeding lines and commercial cultivars, incl. “Old Yella”. The decomposition of the yellow coloration during flower development suggests that the corresponding pigment may be a flavonol compound as observed in cotton (Tan et al., 2013). Our further breeding efforts will focus on stabilizing the yellow pigment.

**Figure 15:**
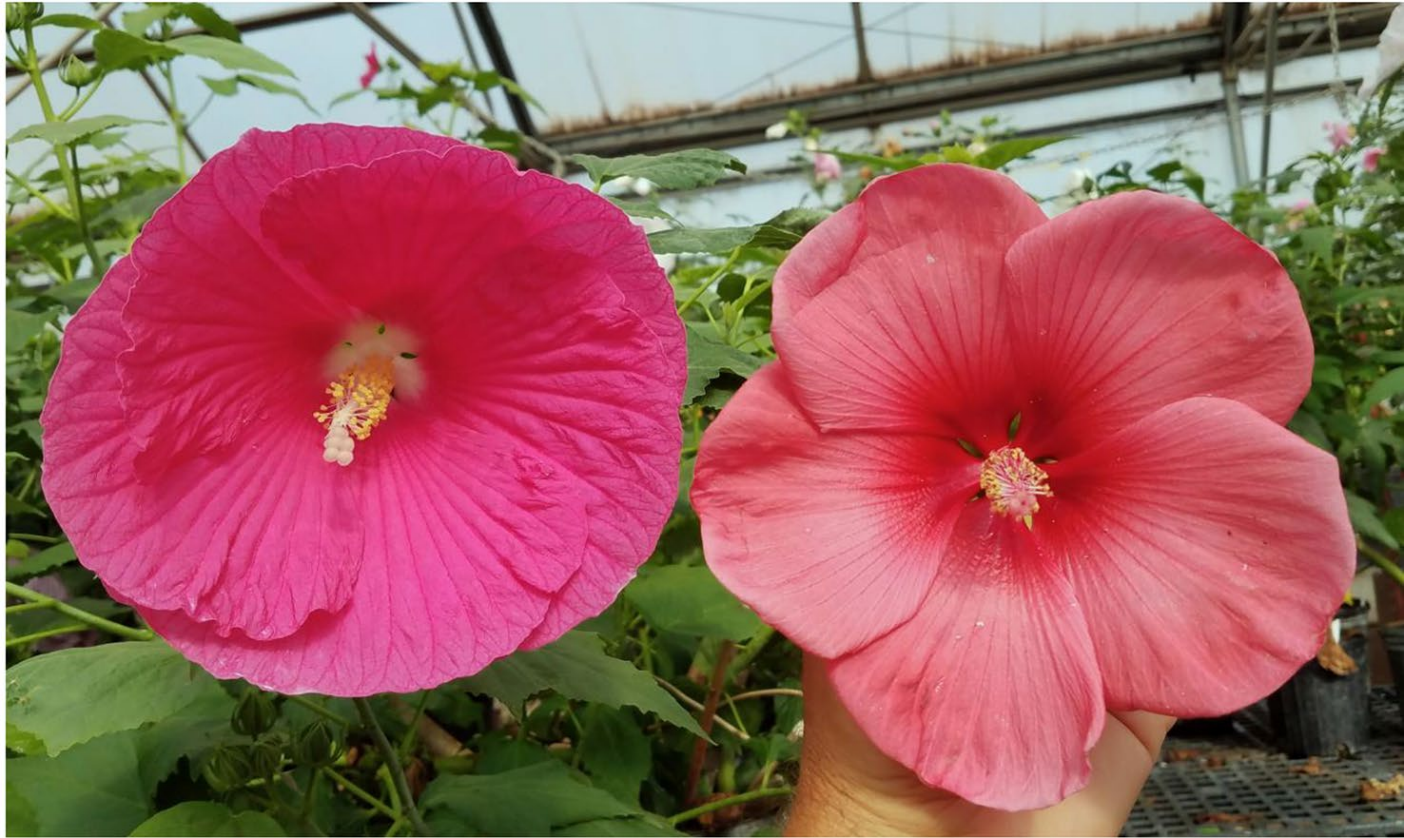
*H.* x *moscheutos* hybrid “180266-M1” (right) with a novel, coral or salmon flower color compared with a pink color flower of the hybrid “TAMUS-4336” (left). Photo credits: Dariusz P. Malinowski.

Although creating yellow flowers in herbaceous winter-hardy hibiscus seems possible, the orange flower color remains a challenging goal. As indicated earlier, orange color in flowers is mainly determined by carotenoids and all winter-hardy hibiscus species in the section *Muenchhusia* lack those pigments. In 2015, we identified a hybrid, commercialized by J Berry Nursery as “Amaretto” in 2018 (Fannin, 2018), with coral or salmon flower color. The flower color of “Amaretto” is very dependent on environmental conditions, such as temperature, light intensity and quality, and humidity; thus, the color intensity may vary. Subsequent breeding resulted in a few selections with a more stable salmon or coral flower color. One of the hybrids, “180266-M1” (**Figure 15**), seems to be a good candidate to focus on in the future efforts to create an “orange”-flowering herbaceous winter-hardy hibiscus hybrid.

## Phenotypic Similarities Between Novel Herbaceous Winter-Hardy Hibiscus Hybrids and Their Tropical Relatives

A molecular analysis of genes responsible for expression of pigments associated with the novel flower colors and expressions of traits associated with flower shapes in our herbaceous winter-hardy hibiscus hybrids and their gene analogs in tropical hibiscus species is beyond the scope of this publication. Although herbaceous winter-hardy hibiscus species and the tropical *H*. *rosa-sinensis* belong to different sections within the *Hibiscus sensu lato* clade (Pfeil et al., 2002), one may speculate that both hibiscus groups had a common ancestor (Fryxell, 1965; Hawkes and Smith, 1965; Fryxell, 1967; Pfeil et al., 2002; Black, 2017) and therefore share common gene families (Kim et al., 2017). By combining genomes of several herbaceous winter-hardy hibiscus species, we have brought together genes of their distant progenitors whose evolutionary life histories were common at one time. Recent advances in genomics suggest that the full metabolic capacity of hybrid organisms has yet to be analyzed, especially the activation of silent metabolism that may result in expression of metabolites and traits previously not known in the immediate parents, but likely present in the near or far evolutionary history of the parentage (Lewinsohn and Gijzen, 2009; Pollier et al., 2011). The numerous common phenotypic flower traits, e.g., colors and shapes, seen in our novel herbaceous winter-hardy hibiscus hybrids and their distant relative *H. rosa-sinensis* hybrids would support the hypothesis of “awakened” silent genes triggering inactive biosynthetic pathways in the multi-interspecific winter-hardy hybrids. We suggest that further intensive hybridization within the *Hibiscus* species in the section *Muenchhusia* will eventually result in creating winter-hardy hibiscus hybrids with more novel flower colors and color combinations, and flower shapes closely resembling the tropical hibiscus. This approach will bypass genetic barriers preventing interspecific hybridization in the two groups in order to create “tropical-like” winter-hardy hibiscus hybrids.

## Conclusion

As outlined in our review, development of interspecific hybrids between herbaceous winter-hardy hibiscus species and the tropical *H. rosa-sinensis* by traditional breeding methods may not be achievable due to the occurrence of multi-level genetic incompatibility reactions. Recent progress in herbaceous winter-hardy hibiscus breeding indicates that broadening genetic variability by intensive interspecific hybridization among hibiscus species in the section *Muenchhusia* has resulted in appearance of novel flower traits, such as new colors and color combinations, and new flower shapes. Frequently, flowers of these hybrids phenotypically resemble flowers of the tropical *H. rosa-sinensis* despite a long history of divergent evolution between these two groups. For scientists, the fundamental question is whether the observed similarities are coincidental and resulting from convergent evolution or if by creating novel gene recombinations we have enabled a transcription of silent genes that have been inherited by winter-hardy hibiscuses from the common ancestor with tropical hibiscus species and may regulate the expression of similar traits. The breeding strategy we applied has resulted in a rapid development of novel flower colors in herbaceous winter-hardy hibiscus and appearing of the first hybrids that closely resemble modern tropical hibiscus hybrids. These novel herbaceous winter-hardy hibiscus hybrids are adapted to temperate environments and will significantly increase the assortment of “tropical-looking” perennial ornamentals in cooler climates.

## Author Contributions

DM and WP conducted breeding of winter-hardy hibiscus hybrids. KY-H evaluated several of the winter-hardy hibiscus hybrids. All authors conceived, wrote individual sections, and approved the final manuscript.

## Funding

The hibiscus breeding project (DM and WE) was partially supported by J Berry Nursery, Grant# M1801321.

## Conflict of Interest

The authors declare that the research was conducted in the absence of any commercial or financial relationships that could be construed as a potential conflict of interest.

## References

[B1] Akpan G. A. (2007). “Hibiscus: *Hibiscus rosa-sinensis*,” in Flower breeding and genetics: Issues, challenges and opportunities for the 21st century. Ed.Anderson N. O. (Dordrecht:Springer), 479–490. 10.1007/978-1-4020-4428-1_17

[B2] Bates D. M. (1965). Notes on the cultivated Malvaceae. 1. Hibiscus. Baileya 13, 57–130.

[B3] Black C. (2017). Did Hibiscus grow in the Jurassic age of dinosaurs? [Internet]. Hidden Valley Hibiscus Newsletter; [cited 2019 Feb 7]. Available from: http://www.hiddenvalleyhibiscus.com/newsletters/february2017.htm.

[B4] Black C. (2019a). The mystery of blue hibiscus flowers. Blue, lavender, purple, and brown hibiscus are not found in the wild. Where Did They Come From? [Internet]. Hidden Valley Hibiscus; [cited 2019 Feb 7]. Available from: http://www.hiddenvalleyhibiscus.com/history/blueflowers.htm.

[B5] Black C. (2019b). The mystery of hibiscus colors. Why do hibiscus flowers change color? [Internet]. Hidden Valley Hibiscus; [cited 2019 Feb 7]. Available from: http://www.hiddenvalleyhibiscus.com/misc/colors.htm.

[B6] Casey S. (1997). Women invent!: Two centuries of discoveries that have shaped our world. Chicago (IL):Chicago Review Press.

[B7] Eeckhaut T. G. R. Van Huylenbroeck J. M. De Riek J. Van Bockstaele E. (2004). Interspecific hybridization between *Hibiscus syriacus* L. and *Hibiscus paramutabilis* Bailey. Acta Hortic. 630, 85–90. 10.17660/ActaHortic.2004.630.10

[B8] Eeckhaut T. (2003). Ploidy breeding and interspecific hybridization in Spathiphyllum and woody ornamentals. [dissertation thesis]. [Gent, Belgium]: University of Gent. Available from: https://biblio.ugent.be/publication/521786/file/1875116.

[B9] Fannin B. (2018). Texas A&M AgriLife’s novel winter-hardy hibiscus hybrids hitting U.S. market. College Station (TX):Texas A&M Communications;. [cited 2019 Feb 7]. Available from: .

[B10] Fryxell P. A. (1965). Stages in the evolution of *Gossypium* L. Advancing Front. Plant Sci. 10, 31–56.

[B11] Fryxell P. A. (1967). The interpretation of disjunct distributions. Taxon 16, 316–324. 10.2307/1216382

[B12] Fryxell P. A. (1980). A revision of the American species of *Hibiscus* Section *Bombicella* (Malvaceae). Tech. Bull. USDA 1624, 1–52.

[B13] Gettys L. A. (2012). Genetic control of white flower color in scarlet rosemallow (*Hibiscus coccineus* Walter). J. Hered. 103, 594–597. 10.1093/jhered/ess009 22569784

[B14] Ha Y.-M. Shim K.-K. Kang H.-C. Lim K.-B. (2014). A new cultivar ‘Tohagol Red’ with unique flower shape and color. Flower Res. J. 22, 278–282. 10.11623/frj.2014.22.4.10

[B15] Ha Y.-M. Lim K.-B. Shim K.-K. (2015). Development of a new hibiscus cultivar ‘Daewangchun’ with vigorous growth and unique red eye through interspecific hybridization. Korean J. Hortic. Sci. Tech. 33, 453–458. 10.7235/hort.2015.14170

[B16] Hawkes J. G. Smith P. (1965). Continental drift and the age of angiosperm genera. Nat. (London) 207, 48–50. 10.1038/207048a0

[B17] Hemming E. S. (1952). The perfect mallow (*Hibiscus*) marvel. Plant Life. 8, 153–154.

[B18] Hemming E. S. (1959). The hardy herbaceous mallows. Natl. Hortic. Mag. 38, 143–144.

[B19] Hinsley S. R. (2009). Malvaceae info. Hibiscus section Muenchhusia [Internet]. Stewart R. Hinsley; [cited 2019 Feb 7]. Available from: http://www.malvaceae.info/Genera/Hibiscus/Muenchhusia.php.

[B20] Hinsley S. R. (2012). Malvaceae info. Chromosome counts for Malvaceae [Internet]. Stewart R. Hinsley; [cited 2019 Feb 7]. Available from: http://www.malvaceae.info/Biology/Chromosomes.php.

[B21] Hogenboom N. (1973). A model for incongruity in intimate partner relationships. Euphytica 22, 219–233. 10.1007/BF00022629

[B22] International Hibiscus Society (2019). Registered and non-registered cultivars [Internet]. International Hibiscus Society; [cited 2019 Feb 7]. Available from: http://www.internationalhibiscussociety.org/SEArchive/A/cvindex1.php?letter=a.

[B23] Justia Patents (2012). Hibiscus plant named ‘Satellite’ PP23,759 [Internet]. Justia Patents; [cited 2019 Feb 7]. Available from: https://patents.justia.com/patent/20120222175.

[B24] Kang H.-C. Ha Y. M. Kim K. H. (2015). A new hibiscus cultivar ‘Woolred’ with vigorous growth and unique flower shape through interspecific hybridization. Flower Res. J. 23, 276– 280. 10.11623/frj.2015.23.4.45

[B25] Karlsson M. G. Heins R. D. Gerberick J. O. Hackmann M. E. (1991). Temperature driven leaf unfolding rate in *Hibiscus rosa-sinensis*. Sci. Hortic. 45, 323–331. 10.1016/0304-4238(91)90078-D

[B26] Kennedy C. S. (1960). Adventures with hardy herbaceous hibiscus. Am. Hortic. Mag. 39, 199–203. https://www.ahsgardening.org/uploads/pdfs/1960-10r.pdf

[B27] Kim Y.-M. Kim S. Koo N. Shin A.-Y. Yeom S.-I. Seo E. (2017). Genome analysis of *Hibiscus syriacus* provides insights of polyploidization and indeterminate flowering in woody plants. DNA Res. 24, 71–80. 10.1093/dnares/dsw049 28011721 PMC5381346

[B28] Kuligowska K. Simonsen M. Lutken H. Muller R. (2013). Breeding of *Hibiscus rosa-sinensis* for garden use in Denmark. Acta Hortic. 990, 235–242. 10.17660/ActaHortic.2013.990.27

[B29] Kuligowska K. Lutken H. Christensen B. Muller R. (2016). Interspecific hybridization among cultivars of hardy *Hibiscus* species section *Muenchhusia*. Breed. Sci. 66, 300–308. 10.1270/jsbbs.66.300 27162501 PMC4785007

[B30] Kuwada H. (1964). The newly artificially raised amphidiploid plant named ‘Ai-Fuyo’ (*Hibiscus mutamoscheutos*), obtained from the progeny of *H. mutabilis* x *H. moscheutos*. Jpn. J. Breed. 14, 27–32. 10.1270/jsbbs1951.14.27

[B31] Kyung H.-Y. Kim J.-H. (2001). Effects of flowering season on the compatibilities of interspecific single, three way, and double crosses among *Hibiscus syriacus*, *H. sinosyriacus*, and interspecific hybrids (*H. syriacus* x *H. paramutabilis*). J. Kor. Soc Hortic. Sci. 42, 568–574.

[B32] Kyung H.-Y. Park S.-M. Kim J.-H. (2001). Effects of bud and old-flower pollination on the interspecific single and three way cross compatibilities between cultivar groups of *Hibiscus syriacus* and *H. sinosyriacus*. J. Kor. Soc Hortic. Sci. 42, 575–580.

[B33] Lawton B. (2004). Hibiscus: Hardy and tropical plants for the garden. Inc. Portland, OR:Timber Press.

[B34] Ledbetter K. (2015). AgriLife Researcher develops a painter’s palette of winter-hardy hibiscus colors. College Station (TX): Texas A&M AgriLife Communications;. [cited 2019 Feb 7]. Available from: https://today.agrilife.org/2015/10/27/agrilife-researcher-develops-a-painters-palette-of-winter-hardy-hibiscus-colors.

[B35] Ledbetter K. (2017). Colorful winter-hardy hibiscus hybrids continue trek to consumer gardens. College Station (TX): Texas A&M AgriLife Communications;. c2017 [cited 2019 Feb 7]. Available from: https://today.agrilife.org/2017/09/15/colorful-winter-hardy-hibiscus-hybrids-continue-trek-consumer-gardens/.

[B36] Lewinsohn E. Gijzen M. (2009). Phytochemical diversity: The sounds of silent metabolism. Plant Sci. 176, 161–169. 10.1016/J.PLANTSCI.2008.09.018

[B37] Linnaeus C. (1753). Species Plantarum Vol. 1 Stockholm:Laurentius Salvius, 694. 10.1016/j.plantsci.2008.09.018

[B38] MacIntyre J. P. Lacroix C. R. (1996). Comparative development of perianth and androecial primordia of the single flower and the homeotic double-flowered mutant in *Hibiscus rosa-sinensis* (Malvaceae). Can. J. Bot. 74, 1871–1882. 10.21273/HORTSCI.47.2.291

[B39] Malinowski D. P. Brown R. S. Pinchak W. E. (2012a). ‘Blue Angel’ winter-hardy hibiscus (*Hibiscus ×moscheutos* L.). HortScience 47, 289–290. 10.21273/HORTSCI.47.2.289

[B40] Malinowski D. P. Brown R. S. Pinchak W. E. (2012b). ‘Robert Brown’ winter-hardy hibiscus (*Hibiscus ×moscheutos* L.). HortScience 47, 291–292. 10.21273/HORTSCI.47.2.291

[B41] Martin C. Gerats T. (1993). Control of pigment biosynthesis genes during petal development. Plant Cell 5, 1253–1364. 10.1139/b96-224 12271025 PMC160358

[B42] Mercuri A. Braglia L. De Benedetti L. Ballardini M. Nicoletti F. Bianchini C. (2010). New genotypes of *Hibiscus × rosa-sinensis* through classical breeding and genetic transformation. Acta Hortic. 855, 201–208. 10.2307/3869778

[B43] Meyerowitz E. M. Smyth D. R. Bowman J. L. (1989). Abnormal flowers and pattern formation in floral development. Development 106, 209–217. 10.17660/ActaHortic.2010.855.29

[B44] Niimoto D. H. (1966). Chromosome numbers of some *Hibiscus species* and other Malvaceae. Baileya 14, 29–34.

[B45] Paek K. Hwang J. Jong S. Park S. (1989). Somatic hybridization by protoplast fusion in *Hibiscus syriacus* and *Hibiscus rosa-sinensis*. Kor. J. Breed. 21, 95–102.

[B46] Palmer K. Palmer M. (1954). Hibiscus unlimited and how to know them. St. Petersburg (FL):Creative Press Inc.

[B47] Pfeil B. E. Brubaker C. L. Craven L. A. Crisp M. D. (2002). Phylogeny of *Hibiscus* and the tribe Hibisceae (Malvaceae) using chloroplast DNA sequences of ndh F and the rp 116 intron. Syst. Bot. 27, 333–350. www.jstor.org/stable/3093875

[B48] Pollier J. Moses T. Goossens A. (2011). Combinatorial biosynthesis in plants: A (p)review on its potential and future exploitation. Nat. Prod. Rep. 28, 1897–1916. 10.1039/C1NP00049G 21952724

[B49] Pounders C. T. Sakhanokho H. F. (2016). ‘Hapa White’, ‘Hapa Pink’, and ‘Hapa Red’ interspecific hybrid hibiscus cultivars. HortScience 51, 1616–1617. 10.1039/c1np00049g

[B50] Puckhaber L. S. Stipanovic R. D. Bost G. A. (2002). “Analyses for flavonoid aglycones in fresh and preserved *Hibiscus* flowers,” in Trends in new crops and new uses. Eds.Janick J. Whipkey A. (Alexandria, VA:ASHS Press), 556–563. 10.21273/HORTSCI11291-16

[B51] Rakhimkhanov Z. B. Sadykov A. S. Ismailov A. I. Karimdzhanov A. K. (1970). A study of the anthocyanins of hibiscus hybrids. Khimiya Prirodnykh Soeclinenii 6 (1), 130. 10.1007/BF00564179

[B52] Rakhimkhanov Z. B. Sadykov A. S. Ismailov A. I. Karimdzhanov A. K. (1973). Anthocyanins of hybrid hibiscuses. Chem. Nat. Comp. 9, 152–155. 10.1007/BF00564179

[B53] Ronse De Craene L. P. (2003). The evolutionary significance of homeosis in flowers: a morphological perspective. Int. J. Plant Sci. 164 (S5), S225–S235. 10.1007/BF00563333

[B54] Salamah A. Prihatiningsih R. Rostina I. Dwiranti A. (2018). Comparative morphology of single and double flowers in *Hibiscus rosa-sinensis* L. (Malvaceae): A homeosis study. Am. Inst. Phys. Conf. Proc. 2023, 020136. 10.1086/376878

[B55] Shirley B. W. Hanley S. Goodman H. M. (1992). Effects of ionizing radiation on a plant genome: analysis of two Arabidopsis transparent testa mutations. Plant Cell 4, 333–347. 10.1063/1.5064133 1354004 PMC160133

[B56] Shobha K. S. Selvaraj Y. Bhat R. N. (1999). Pigmentation studies in *Hibiscus rosa-sinensis* cultivars. Indian J. Hortic. 56, 179–183. 10.1105/tpc.4.3.333

[B57] Shu M. J. (2007). *Hibiscus* Linnaeus. Flora China 12, 286–294. http://flora.huh.harvard.edu/china//PDF/PDF12/Hibiscus.pdf

[B58] Singh F. Khoshoo T. N. (1970). Chromosomal polymorphism within the *Hibiscus rosa-sinensis* complex. Caryologia 23, 19–27. 10.1080/00087114.1970.10796359

[B59] Tan J. Wang M. Tu L. Nie Y. Lin Y. Zhang X. (2013). The flavonoid pathway regulates the petal colors of cotton flower. PloS One 8 (8), e72364. 10.1080/00087114.1970.10796359 23951318 PMC3741151

[B60] Thomson L. A. J. Braglia L. (2019). Review of Fiji Hibiscus (Malvaceae-Malvoideae) species in section Lilibiscus. Pac. Sci. 73, 79–121. 10.1371/journal.pone.0072364

[B61] United States Plant Patent (2003a). US PP13,734. Hibiscus plant named ‘Angelique’ [Internet]. Google Patents; [cited 2019 Feb 07]. Available from 10.2984/73.1.5 https://patentimages.storage.googleapis.com/4c/06/c3/a88f185eb555a4/USPP13734.pdf.

[B62] United States Plant Patent (2003b). US PP13,630. Hibiscus plant named ‘Old Yella’. [Internet]. Google Patents; [cited 2019 Feb 07]. Available from: https://patentimages.storage.googleapis.com/9c/aa/f7/09eb677362cfca/USPP13630.pdf.

[B63] United States Plant Patent (2003c). US PP13,751. Hibiscus plant named ‘Pink Comet’. [Internet]. Google Patents; [cited 2019 Feb 07]. Available from: https://patentimages.storage.googleapis.com/73/50/3c/20d66cf0d69248/USPP13751.pdf.

[B64] United States Plant Patent (2006). US PP16,669. Hibiscus plant named ‘Cherub’. [Internet]. Google Patents; [cited 2019 Feb 07]. Available from: https://patentimages.storage.googleapis.com/91/82/de/be8058fa649d2a/USPP16669.pdf.

[B65] United States Plant Patent (2012). Hibiscus plant named ‘New Old Yella’. Pub. No. US 2012/0222181 P1. [Internet]. Google Patents; [cited 2019 Feb 07]. Available from: https://patentimages.storage.googleapis.com/7c/5c/97/d04a54c0c9617d/US20120222181P1.pdf.

[B66] Van Huylenbroeck J. M. De Riek J. De Loose M. (2000). Genetic relationships among *Hibiscus syriacus*, *Hibiscus sinosyriacus* and *Hibiscus paramutabilis* revealed by AFLP, morphology and ploidy analysis. Gen. Res. Crop Evol. 47, 335–343. 10.1023/A:1008750929836

[B67] Van Laere K. Van Huylenbroeck J. M. Van Bockstaele E. (2007). Interspecific hybridisation between Hibiscus syriacus, *Hibiscus sinosyriacus* and *Hibiscus paramutabilis*. Euphytica 155, 271–283. 10.1007/s10681-006-9328-8

[B68] Van Tuyl J. De Jeu M. (1997). “Methods for overcoming interspecific crossing barriers,” in Pollen biotechnology for crop production and improvement. Eds.Shivanna K. Sawhney V. (Cambridge, UK: University Press), 273–292. 10.1023/A:1008750929836

[B69] Vazquez-Thello A. Yang L. J. Hidaka M. Uozumi T. (1996). Inherited chilling tolerance in somatic hybrids of transgenic *Hibiscus rosa-sinensis* x transgenic *Lavatera thuringiaca* selected by double-antibiotic resistance. Plant Cell Rep. 15, 506–511. 10.1007/s10681-006-9328-8 24178462

[B70] Wang X. (2010). The dawn angiosperms: Uncovering the origin of flowering plants. Berlin:Springer Verlag. 10.1017/CBO9780511525469.015

[B71] Wilcox E. V. Holt V. S. (1913). Ornamental *Hibiscus* in Hawaii. Hawaii Agric. Res. Exp. Sta. Bull. 29, 65. 10.1007/BF00232983

[B72] Winters H. F. (1970). Our hardy *Hibiscus* species as ornamentals. Econ. Bot. 24, 155–164. 10.1007/978-3-642-01161-0

[B73] Yu T. Y. Yeam D. Y. Kim Y. (1976). A study on the breeding of *Hibiscus syriacus* L. @ on hybridisation among introduced tetraploids and *H. rosa-sinensis*. J. Korean Soc Hortic. Sci. 17, 107–112.

[B74] Zhao D. Tao J. (2015). Recent advances on the development and regulation of flower color in ornamental plants. Front. Plant Sci. 6, 261. 10.1007/BF02860595 25964787 PMC4410614

[B75] Zhurenko L. (2019).‘Serdse Matery’ (Mother’s heart) hibiscus picture. Available from: https://www.facebook.com/photo.php?fbid=2413478198886599&set=pb.100006733273681.-2207520000.1569530464.&type=3&theater.

